# Flicker-Suppressed Neuromorphic Unit for Dynamic Vision Processing

**DOI:** 10.1021/acsnano.5c18939

**Published:** 2026-02-25

**Authors:** Pengshan Xie, Shuhui Shi, Lei Ran, Chunhua Wang, Dengji Li, Yuxuan Zhang, Yiyang Wei, Quan Quan, Bowen Li, You Meng, Weijun Wang, Boxiang Gao, Changyong Lan, Michael K. H. Leung, Zhongrui Wang, Johnny C. Ho

**Affiliations:** † Department of Materials Science and Engineering, 53025 City University of Hong Kong, Kowloon 999077, Hong Kong SAR; ‡ School of Microelectronics, 255310 Southern University of Science and Technology, Shenzhen 518055, China; § School of Energy and Environment, City University of Hong Kong, Kowloon 999077, Hong Kong SAR; ∥ School of Optoelectronic Science and Engineering, 12599 University of Electronic Science and Technology of China, Chengdu 611731, China; ⊥ Shenzhen Research Institute, City University of Hong Kong, Shenzhen 518057, China; # State Key Laboratory of Terahertz and Millimeter Waves, City University of Hong Kong, Kowloon 999077, Hong Kong SAR; ¶ Institute for Materials Chemistry and Engineering, Kyushu University, Fukuoka 816-8580, Japan

**Keywords:** flicker-suppressed, dynamic vision processing, heterojunction, reservoir computing, channel stress, ultrafast stimulation

## Abstract

Inspired by the dynamic visual perception of flying insects, rapid collision warning systems are crucial for advancing autonomous driving and machine control. Although neuromorphic devices show significant potential for replicating insect vision systems, they are hindered by limitations in the sensing frequency, signal-to-noise ratio, and flicker noise. Here, we use a combination of a homojunction and heterojunction to emulate the two different transmission modes of nerve signals via gate-voltage modulation. The structural design and heterojunction effects enabled artificial neurons to respond to high-frequency visible-light signals and achieve an information transmission rate of 2100 bits s^−1^. By connecting the leaky integrate-and-fire neural device in series with the synaptic device, we successfully generated action potentials and postsynaptic potential responses, significantly reducing cumulative threshold flicker noise. Using in-sensor reservoir computing, we achieved trajectory recognition across four car orientations with an optimized training process, providing valuable insights into device design and applications in visual bionics.

## Graphical abstract

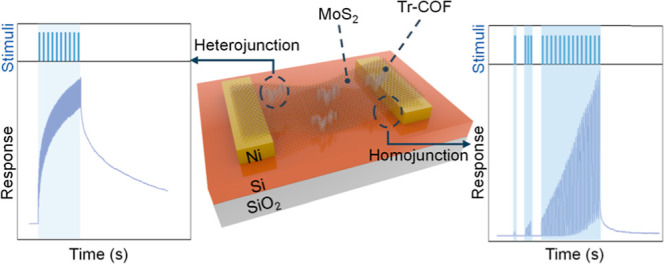

## Introduction

Biological vision systems, refined through long-term evolutionary optimization, enable organisms to extract survival-critical spatiotemporal features via phototransduction and neuromodulation pathways.^1,2^ To overcome biological vision’s limitations, machine vision, inspired by these biological systems, has emerged as a rapidly advancing field. When integrated with artificial intelligence, machine vision systems can seamlessly merge spatial and temporal data streams.^3,4^ In real-time applications that demand swift recognition, judgment, and decision-making, there is a continual need to enhance these systems’ multimodal perception capabilities to navigate complex environments and make quick determinations amidst various interferences.^5,6^

One significant challenge in machine vision is flicker noise, a low-frequency phenomenon in which the spectral density is inversely related to frequency.^7^ This noise can cause irregular brightness fluctuations in images, reducing resolution and impairing image processing algorithms. Therefore, mitigating flicker noise is crucial for improving the performance and stability of systems such as autonomous driving and surveillance under complex lighting conditions.^8,9^ Neural computation relies on two complementary signaling modalities: axonal electrical transmission and synaptic chemical transduction.^10^ These modes facilitate rapid and efficient information transmission within the nervous system, coordinating perception, movement, and complex physiological and cognitive functions.^11,12^ Flying insects exemplify this efficiency with their highly refined visual structures. Their compound eyes, composed of thousands of ommatidia, achieve refresh rates of 250–300 Hz, significantly faster than human vision.^13^ This enables them to process visual information with exceptional temporal and spatial resolution, allowing for rapid identification, recognition, and response. In intelligent driving, especially at high speeds, effective collision avoidance and route planning are constrained by physical hardware limitations. The von Neumann architecture struggles to handle the vast influx of data from multisensor arrays, such as lidar, millimeter-wave radar, and general cameras. This leads to fundamental bottlenecks in current autonomous systems.^14^ To overcome these challenges, there is a need for cooptimization of photonic sensing frontends and neurosynaptic backends. Signal filtering properties, crucial for the complete conduction of neural signals, are vital. The nervous system must effectively manage noise and interference from both external and internal sources to ensure clear, accurate, and effective information processing. Achieving tight photon-to-spike coupling can circumvent the energy demands of digital noise cancellation in conventional systems while preserving essential features for collision prediction.

In this work, we introduce a bioinspired flicker-noise-reduced real-time in-sensor visual system (FRIV) that replicates the entire neural pathway from phototransduction to motor encoding. We developed a bioinspired neuromorphic platform by integrating a π-conjugated triazine-based covalent organic framework (Tr-COF) with suspended few-layer molybdenum disulfide (MoS_2_) channels to emulate axonal and synaptic signaling processes. With its porous architecture, Tr-COF induces a photogating effect under optical excitation, mimicking postsynaptic potential transmission. Meanwhile, suspended source/drain electrodes with adjustable air gaps enable gate-voltage-modulated action potential propagation, resulting in homojunction and replicating axonal signal dynamics. Our systematic analysis demonstrated that leaky integrate-and-fire (LIF) neuronal behavior could be precisely controlled by wavelength-dependent photostimulation and geometric optimization of the air gap. By cascading two different device architectures, we comprehensively simulated neural signal transmission pathways, showcasing perceptual encoding and threshold-based filtering that effectively suppresses flicker noise.

This visual system enabled a neuromorphic response to 1000 Hz visible-light signals, driven by the synergistic effects of the structural design and heterojunctions. It achieved an information transmission rate of 2100 bits s^–1^. Notably, the top ion-gate configuration exhibited ultrasteep switching characteristics (subthreshold swing <150 mV dec^–1^) across varying gate distances, highlighting different spatial dependencies. Leveraging these attributes, the artificial neurons facilitate high-efficiency in-sensor storage and reservoir computing (RC), enabling streamlined training processes for tasks such as tracking the trajectory of a moving car.

## Results and Discussion

Insects possess highly efficient dynamic vision systems optimized for motion perception in rapidly changing environments. Visual signals captured by compound eyes are processed through a hierarchical lamina–medulla–lobula pathway, in which early-stage noise suppression and contrast conditioning are followed by spatiotemporal integration and motion-selective processing (Figure 1a).^15^ This layered organization enables insects to extract motion-relevant features directly from continuous luminance changes rather than from frame-based image reconstruction. A commonly used quantitative indicator of the temporal processing capability of insect vision is flicker fusion frequency (FFF), which reflects the maximum frequency at which luminance modulation can be resolved as a continuous signal. Owing to the compound eye architecture and efficient neural processing, the FFF of flying insects such as mosquitoes and drosophila can reach approximately 250–300 Hz. Importantly, while FFF does not represent the full complexity of insect motion-processing pathways, it provides an intuitive benchmark for the temporal resolution and noise tolerance required for artificial visual systems targeting agile motion perception.^16^ The neural activity of organisms can be roughly divided into two parts: potential changes in neuronal axons and intersynaptic transmitter release. When a neuron is stimulated, the sodium channel in its membrane opens, allowing sodium ions (Na^+^) to rapidly enter the cell, leading to depolarization and formation of an action potential. Immediately afterward, potassium channels open, and potassium ions (K^+^) flow out of the cell, returning the membrane potential to a resting state. The action potential will propagate down the axon, similar to how “current” travels along a wire. Through this change in potential, signals can be rapidly conducted from the cell body of a neuron to its end (Figure 1bi).^17^ The LIF model correspondingly describes the process of membrane potential changes in neurons (Figure 1bii).^18^ At the synapse, electrical signals are converted to chemical signals, and neurotransmitters are released into the synaptic gap, where they bind to the next neuron, generating changes in conductance and accumulation of potential, triggering subsequent neural activity (Figure 1c).^19^ The simulation of the whole process of neural activity allows for more efficient information transfer. Threshold-triggering mechanisms can prevent information channels from being occupied by redundant or useless messages.

**Figure 1:**
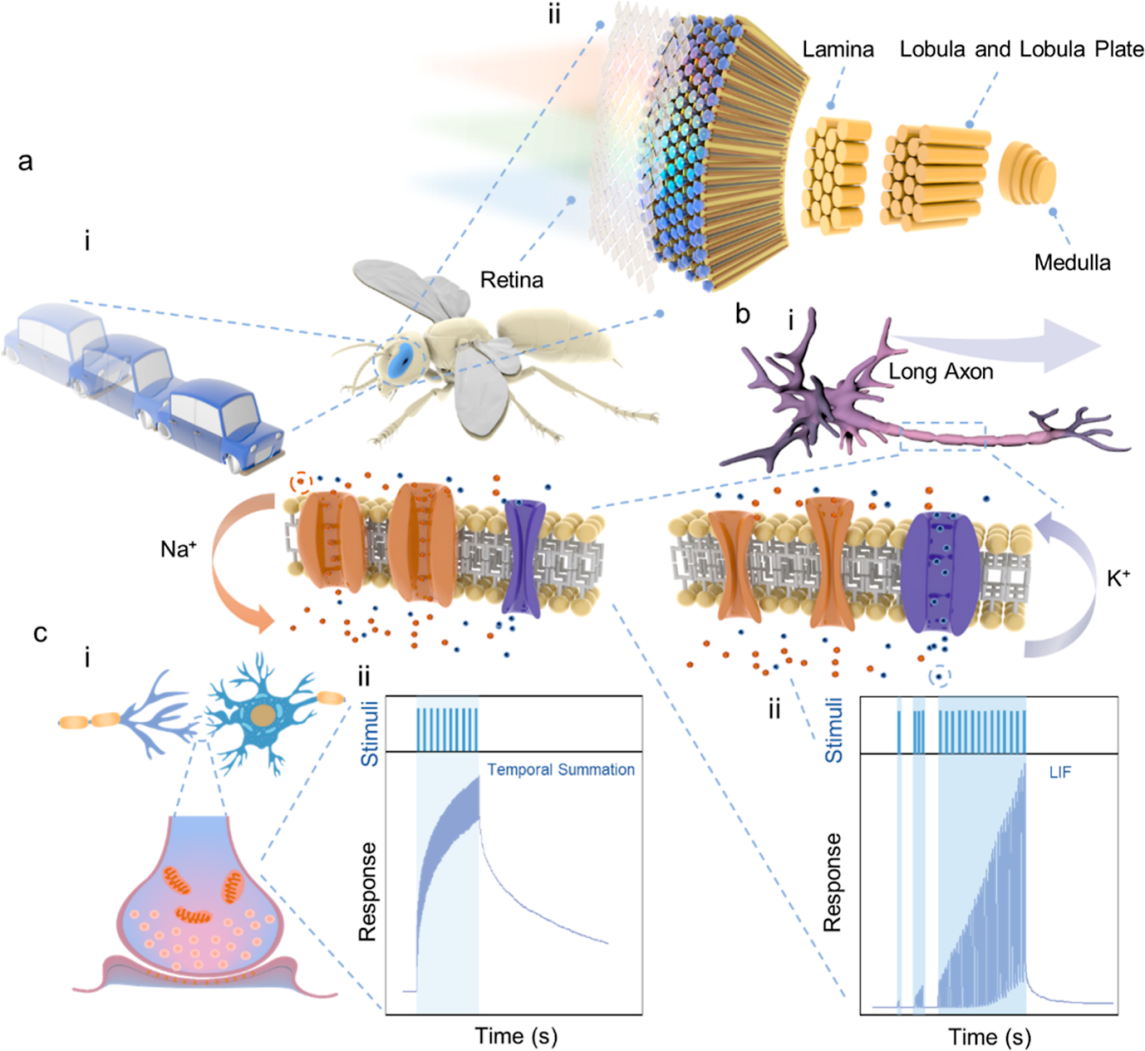
Full agile perception process inspired by the flying insects’ vision system. (a) Specific construction of flying insect vision. (b) (i) Schematic diagram of a neuron’s structure and the action potential generation process. (ii) Corresponding LIF neuron response of action potential current under continuous stimulus. (c) (i) Schematic diagram of the structure of a synapse, including the presynaptic membrane, the postsynaptic membrane, and the neurotransmitters. (ii) Corresponding synaptic response of postsynaptic potentials.

Due to their good optoelectronic properties, two-dimensional materials provide a versatile platform for emulating the full functionality of neurons. Here, we introduced a few layers of MoS_2_ as the channel, combining them with the Tr-COF and structural engineering to realize high-FFF synaptic perception and LIF neurons. Our previous work synthesized the porous and laminated Tr-COF.^20^ Owing to the planar structure of building blocks, the high π–π interactions between the adjacent layers were guaranteed, obtaining highly crystalline Tr-COF (Supporting Information Figure 1a). To verify the morphology, scanning and transmission electron microscopy (SEM/TEM) were employed. The typical TEM image of Tr-COF is shown in Supporting Information Figure 1b, revealing abundant pores in the COF network. In addition, as shown in the high-resolution TEM (HRTEM) image, the observed interplanar spacing of 0.363 nm can be indexed to the (001) plane of hexagonal Tr-COF (Supporting Information Figure 1c). Moreover, the SEM images and corresponding energy-dispersive spectroscopy (EDS) mapping images reveal a uniform distribution of C and N elements within the COF network (Supporting Information Figure 2). The powder X-ray diffraction (XRD) pattern was employed to assess the crystallinity of the Tr-COF sample, and the observed several sharp diffraction peaks indicate its fine crystalline nature (Supporting Information Figure 3). Specifically, the diffraction peaks at 4.0°, 12.1°, and 14.7° are ascribed to the (100), (110), and (130) planes, respectively. In contrast, the diffraction peak at about 25° corresponds to π–π stacking interactions between adjacent layers and to the (001) plane in the COF. Moreover, Fourier transform infrared (FTIR) spectroscopy was used to investigate the microstructure of the Tr-COF. As shown in Supporting Information Figure 4, the two observed typical vibration bands at 1620 and 1400 cm^–1^ are attributed to ν­(–N=C−) in the imine bond and pyridine ring, respectively. These results indicate the successful formation of highly crystalline, porous Tr-COF frameworks. For device fabrication, the Tr-COF dispersed in ethanol was spin-coated onto the MoS_2_ channel surface, where a few-layer MoS_2_ channel was prepared by mechanical exfoliation and physical transfer (Figure 2a). Atomic force microscopy (AFM) and Raman spectroscopy both verified the layers and the homogeneity of the MoS_2_ channel (Supporting Information Figures 5 and 6a).^21^ When light stimuli are applied in the channel, electron–hole pairs would be effectively separated at the interface under the effect of energy bands (Figure 2b and Supporting Information Figure 6b).^20,22^ The highly ordered multilayer pore structure of Tr-COF can promote effective transmission and diffusion of photons within the material. Moreover, the out-of-plane π-conjugate structure of Tr-COF results in lower transport barriers for carriers in the out-of-plane direction, thereby tending to transfer carriers away from the channel and to impede recombination at the interface (Figure 2ci).^23,24^ In-plane (3.616) and out-of-plane (1.236) effective mass density functional theory (DFT) calculations verify that carriers have a higher mobility along the out-of-plane direction (Figure 2cii). Longer photogenerated hole lifetimes enable the photogating effect of Tr-COFs, leading to a longer photocurrent relaxation time in the MoS_2_ channel.^25,26^ The diminished signal of the interface observed by photoluminescence (PL) mapping further validates the carrier transfer phenomenon occurring between MoS_2_ and Tr-COF (Supporting Information Figure 7).^27^ In visual perception, the appropriate perception band is also an important prerequisite for applications. An approximately 1.8 eV band gap enables the device to be detected in the visible range.

**Figure 2:**
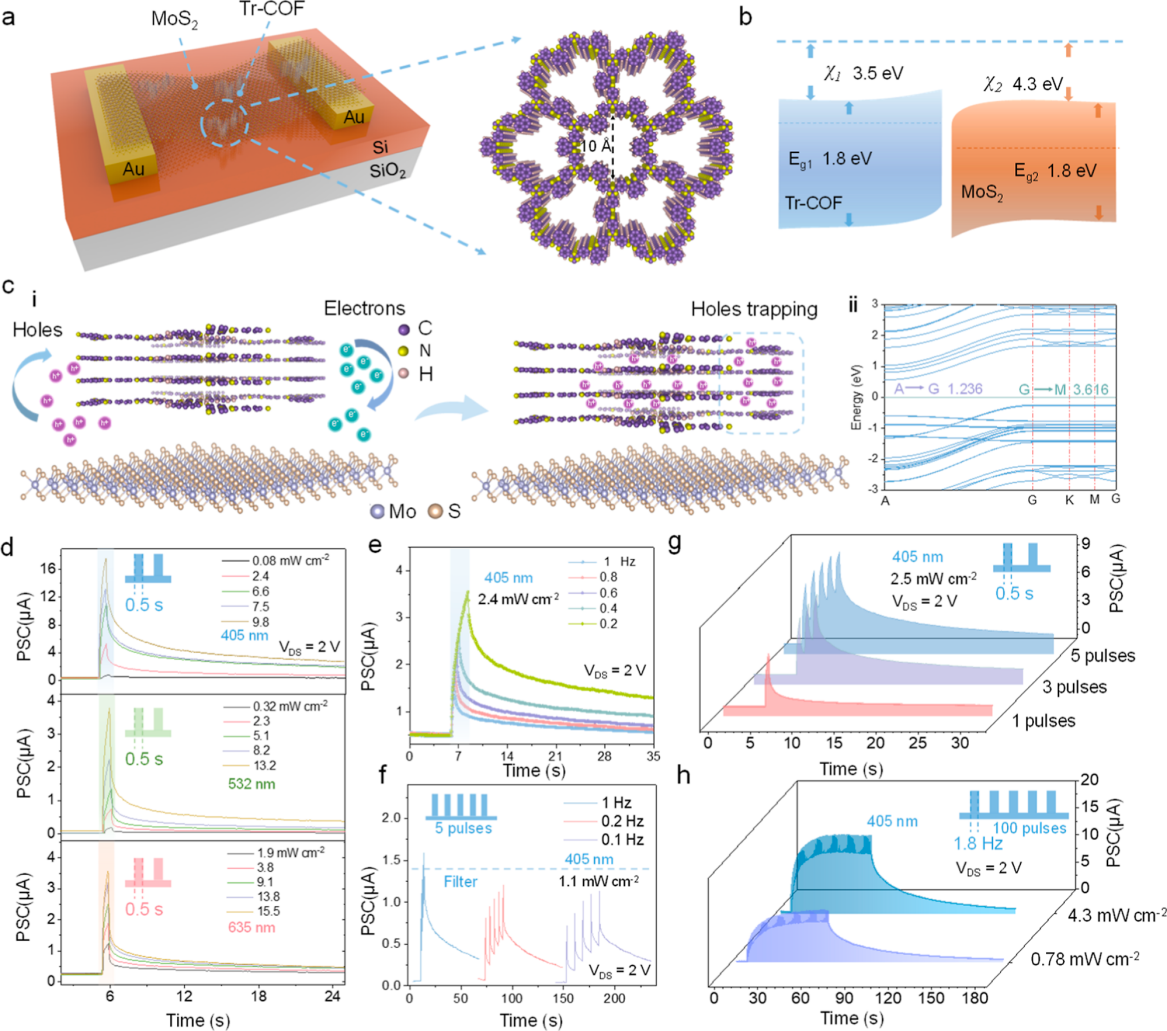
Optoelectronic properties of artificial visual devices for postsynaptic potentials. (a) Schematic diagram of a device structure with suspended structures and Tr-COF. (b) Band structure of the MoS_2_ and Tr-COF heterojunction. (c) (i) The photogating effect induced by the laminar structure of Tr-COF. (ii) In-plane and out-of-plane effective mass DFT calculations. (d) EPSC of the device under visible range stimuli. The spike-intensity-dependent plasticity was observed across different light power densities and a fixed irradiation time (0.5 s). (e) SDDP characteristics of the device under 405 nm light with varying irradiation times. (f) SFDP characteristics of the device. Different frequencies of the same number of light stimuli produced different degrees of conductance accumulation, mimicking the nervous system’s filtering properties. (g) SNDP characteristics of the device. (h) LTP characteristics of the device with 100 continuous stimuli. Different stimulus intensities (light power densities) enabled different saturated conductances.

Supporting Information Figures 8–11 demonstrate the photoresponse of the same device before and after combining with Tr-COF. It is clear that the device successfully transitioned the response mode from the photodetector to the neuromorphic device. Specifically, Tr-COF enhanced the device’s performance, including photocurrent and detectivity. On the other hand, the photogating effect enabled the device to exhibit the temporal summation characteristic of graded neurons. The photoresponse of the standalone Tr-COF also demonstrates the functionality of the heterojunction (Supporting Information Figure 12). A series of essential neural processes that are transmitted between synapses, including excitatory postsynaptic current (EPSC) (Figure 2d), spike duration-dependent plasticity (SDDP) (Figure 2e), spike frequency-dependent plasticity (SFDP) (Figure 2f), and spike number-dependent plasticity (SNDP) (Figure 2g), were successfully realized.^28^ Among them, the corresponding 532 and 635 nm wavelength postsynaptic responses are demonstrated in Supporting Information Figures 13–15. By comparing responses across different bands, the FRIV device showed wavelength-selective properties, with an enhanced EPSC response to short-wavelength optical stimuli. Long-term potentiation (LTP) is the biological basis of learning and memory, enabling neural network plasticity and information encoding and signaling through rapid neural weight inheritance of synaptic connections.^29,30^ The device demonstrated effective LTP characteristics in the visible band with different light power densities (Figure 2h and Supporting Information Figure 16). Correspondingly, paired-pulse facilitation (PPF), a hallmark of short-term synaptic plasticity, mimics the influx of calcium ions that accumulate in the presynaptic membrane in response to two consecutive stimuli.^31^ Supporting Information Figures 17–19 display the different PPF characterizations of the visual devices with visible range light irradiation.

Overall, introducing a Tr-COF/MoS_2_ heterojunction effectively enhanced the device’s optoelectronic performance, resulting in a responsivity (R) of 1.86 × 10^4^ A W^−1^ (Supporting Information Figure 20).^32^ Moreover, the photogating effect extended the carrier lifetime in the MoS_2_ channel, enabling the simulation of postsynaptic potentials.

Rapid visual perception plays an important role in flying in a swarm or avoiding predators. Due to the high photoelectric conversion efficiency of the Tr-COF and MoS_2_ heterojunction, the visual device exhibited postsynaptic potential responses to high-frequency visible light pulses (Figure 3a–c). Stimulated by 1000 Hz light pulses, the device realized effective conductance accumulation with one and three pulses. The insets of Figure 3a–c show details of three consecutive pulses of neuromorphic response on the conductance state. When 1000 Hz continuous pulses were applied to the device, the EPSC increased gradually and then saturated. The continuously enhanced photogating effect maintains the channel’s carrier concentration and gradually stabilizes. Understanding LTP characteristics under high-frequency stimulation is an important prerequisite for the recognition of fast-moving objects. The frequency domain signal-to-noise ratio (SNR) measures the clarity and interference of a signal in the frequency domain.^33^ The conversion of EPSC time to the frequency domain was accomplished by the Fourier transform, and the distribution of SNR about frequency is shown in Figure 3d (Supporting Information Note I). Subsequently, the information transmission rate (*r*), a parameter characterizing the amount of information transmitted per unit of time, was extracted from the frequency-dependent SNR using the Shannon equation (Supporting Information Note II). For 405 nm wavelength inputs with a power density of 11.6 mW cm^–2^, the extracted r is 2100 bit s^–1^. The high r value demonstrates efficient intersynaptic information transfer, suggesting the potential for agile motion perception. To evaluate the ability to encode temporal information, the 5 bit encoding process with the graded changes is shown in Figure 3e. The digital “1” and “0” represent with and without 3.1 mW cm^–2^ 405 nm wavelength irradiation (50 Hz).^34^ The EPSC is highly dependent on the sequence of light irradiation, revealing separated responses to different five-frame actions during the encoding process. The right two figures show the typical encoding process for “10101” and “11011”, respectively.

**Figure 3:**
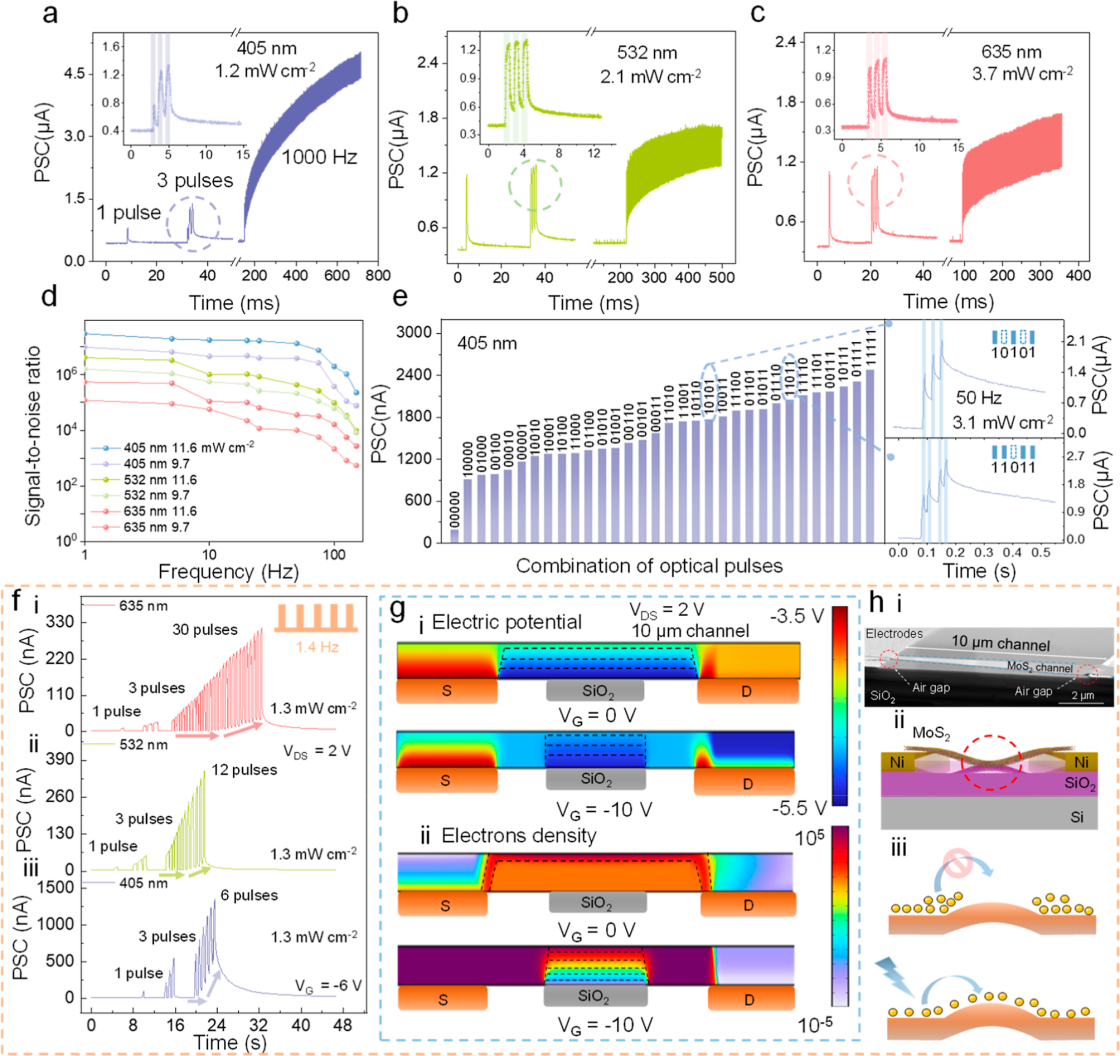
Ultrafast signal sensing and modulation for full-process artificial neurons. EPSC of the FRIV device under (a) 405 nm, (b) 532 nm, and (c) 635 nm 1000 Hz inputs. The insets show the EPSCs from three light pulses. (d) Signal-to-noise ratio with different wavelengths and power intensities. (e) Responses to five-bit light inputs. The inset shows the encoding process for “10101” and “11011” stimulation, where “1” and “0” represent 3.1 mW cm^–2^ light illumination and dark conditions, respectively. (f) LIF neuron response to (i) 635 nm, (ii) 532 nm, and (iii) 405 nm light under V_G_ = −6. At the same power density, the FRIV device exhibited different trigger thresholds across different wavelengths of light. (g) COMSOL simulation of (i) channel electric potential and (ii) electron density. (h) (i) SEM image of the suspended FRIV device channel with S/D air gap. (ii) Schematic cross-section of a suspended structure. (iii) Homogeneous transport barriers formed by suspended structures under negative *V*_G_ modulation.

When gate voltage (*V*_G_) was applied to the FRIV device, the response mode was transferred from the intersynapse to the axons. The LIF neuron effectively models the action potential’s threshold accumulation and release process.^35^ Specifically, the soma would integrate the potential information the postsynaptic membrane receives and trigger an action potential when it reaches the threshold. The action potential is transmitted along the axon through the depolarizing and repolarizing processes of Na^+^ inflow and K^+^ outflow. It allows the membrane potential to return to the resting state. A limited number of light stimuli do not enable the accumulation of conductance to produce action potentials under *V*_G_ modulation. As shown in Figure 3fi–iii, unlike the intersynaptic response, a single light pulse in the visible range can only generate a small action potential current (*I*_AP_) and quickly return to a resting state with *V*_G_ = −6 V. As the number of pulses increases, the conductance begins to accumulate. It fails to revert to its initial state and eventually reaches the trigger threshold.

When *V*_G_ impacts the MoS_2_ channel, the source/drain (S/D) ends’ structures significantly affect the channel’s conductance change.^36,37^ We used finite element methods to simulate the potential distribution and carrier concentration using the COMSOL Multiphysics semiconductor module’s steady-state analysis method. In the suspended structure, MoS_2_ is not in contact with the SiO_2_ insulating layer at the S/D junctions. Therefore, with negative *V*_G_ applied, the MoS_2_ channel in contact with the SiO_2_ insulating layer will have a higher potential energy than that in contact with air because of the different dielectric constants (Figure 3gi).^38^ The electron concentration profile across the channel is quantitatively mapped in Figure 3gii, with the corresponding structural characterization provided by SEM in Figure 3hi. The SEM image clearly reveals an engineered air gap at the S/D interfaces in the 10 μm channel-length device, demonstrating the successful fabrication of the suspended architecture. The cross-sectional schematic of the device is illustrated in Figure 3hii. The change in the energy band structure due to strong capacitive coupling introduces barriers in the homogeneous channel, mimicking the potential accumulation process of LIF neurons (Figure 3hiii). The extra energy from the photons allows carriers to accumulate and eventually cross the potential barriers, leading to sufficiently pronounced accumulation of *I*_AP_ and a corresponding increase in conductance. Therefore, short-wavelength light stimulation can more easily reach the excitation state with the same power density (Figure 3f).

The *V*_G_ regulation in LIF neurons affects the sensitivity of the K^+^ and Na^+^ channels. We explored the effect of different *V*_G_ on the FRIV device’s response to visible-range inputs (Supporting Information Figures 21 and 22). It is manifest that the barrier height is effectively regulated by *V*_G_. In particular, for 532 and 635 nm inputs, *V*_G_ can modulate intersynaptic and axonal signaling modalities. Moreover, after the conductance is accumulated and triggered, the I_AP_ changes more linearly, improving the computational efficiency. Then, we further investigated the effect of the S/D air gap on device performance, which is directly related to the channel length. As a first step, we used a top-contact device structure to eliminate the air gap at the S/D junctions. In this case, *V*_G_ modulation of the device is significantly reduced even when a −20 V gate bias is applied (Supporting Information Figure 23). The 532 nm light pulses make it difficult to realize the LIF neuron response with the S/D bottom-gate, top-contact device structure. The overall decrease in EPSC is mainly due to channel-interface scattering and depletion effects from a larger negative *V*_G_. Similarly, using longer-wavelength (635 nm) light irradiation, LIF neuron responses were still not achieved at *V*_G_ = −10 V (Supporting Information Figure 24). Subsequently, after verifying the effect of the air-gap structure, we adjusted the channel length to control the magnitude of the S/D air gap in the bottom-gate bottom-contact structure. Supporting Information Figure 25 shows an SEM image of the device with a 2 μm channel. It is difficult for *V*_G_ to work on the channel in this situation given the very limited contact with the dielectric layer. *I*_AP_ has almost no obvious changes with *V*_G_ changing from 0 to −10 V (Supporting Information Figure 26). As the channel length was further increased to 5 and 10 μm, the device began to realize intersynaptic and axonal response transitions (Supporting Information Figures 27 and 28). Supporting Information Figure 29 summarizes the device’s response with different channel lengths under the same test conditions. The longer the channel, the more area of MoS_2_ contacts the dielectric layer, resulting in a wider potential range. Meanwhile, under low-temperature conditions, the MoS_2_/Tr-COF heterojunction also exhibits highly efficient carrier separation, resulting in a significant increase in photoconductivity (Supporting Information Figure 30). The voltage-gated transmission barrier also enables neuromorphic response switching at low temperatures, demonstrating the decisive roles of MoS_2_’s structural design and the heterojunction.

The gate-voltage modulation mimics the accumulation and excitation of electric potentials at the ends of nerve membranes. Barrier modulation in homogeneous channels opens the door to the realization of complex neural functions. Therefore, it is crucial to correlate conductance changes under different gate-voltage modulations, with the potential transport arising from ion-channel switching. In situ photocurrent mapping was introduced here to verify the effect of potential barriers on the photocurrents. As shown in Figure 4ai–iii, with increasing negative *V*_G_, the photocurrent in the channel was shifted by the barrier and moved toward one electrode. This intermediate pinch-off and offset toward the electrode response are consistent with our previous simulations. Transfer characteristic curves with different *V*_G_ ranges of the FRIV device were tested and shown in Figure 4b. The clockwise hysteresis indicates the carrier-trapping and release process at the interface.^39^ Moreover, the hysteresis window increases with increasing *V*_G_ scanning range, implying that *V*_G_ directly modulates the carrier transport behavior. Specifically, the application of negative *V*_G_ establishes a gate-tunable potential barrier at the MoS_2_–dielectric interface, enabling electron accumulation on one side. Concurrently, hole trapping at the dielectric–semiconductor interface creates a complementary carrier-modulation mechanism under voltage bias. Therefore, negative *V*_G_ pulses initially decreased the channel current, but after the pulse was withdrawn, the channel current rose rapidly (Figure 4c,d). This multimodal perception provides the potential for devices to simulate complex neural processes. The transient receptor potential vanilloid 1 (TRPV1) channel is a nonselective cation channel that primarily senses heat and capsaicin by conducting calcium and sodium ions.^40^ We used an electrical stimulus on *V*_G_ representing hot water (−10 V, 1 s) and a light stimulus representing capsaicin (405 nm, 0.9 mW cm^–2^, 0.5 s) to simulate the response of TRPV1 (Figure 4e). If hot water was taken first and then exposed to capsaicin, little change in current response was produced. However, if reversed, a significant current enhancement would be observed, which is strongly associated with heat-dependent TRPV1 channels in the presence of capsaicin.^41^ This process validates the feasibility of biomimetic devices to achieve bioreceptor cascade activation and reveals a temporal hierarchy mechanism for multimodal stimulation. The temporally dependent current response pattern accurately mimics the core function of TRPV1 channels in the integration of injurious stimuli. When endogenous capsaicin analogs are released by tissue injury, local temperature elevation significantly enhances injury signaling through a synergistic gating mechanism that underlies the amplification of inflammatory pain.^42^ We also attempted to modulate the device conductance with ions to more closely match the neural modulation process.^43^ Five planar gates with different distances from the channel obtain different hysteresis windows by modulating the ion movement rate in the ion gate (Supporting Information Figure 31). The farther ions spread to form the electronic double layer (EDL), the more obvious the hysteresis of the device and the more noticeable the change in EPSC (Supporting Information Figure 32).^44^ This ion-mediated photoelectrical coupling regulation enables voltage-input-position-dependent modulation of the conductive state (Supporting Information Figure 33). Due to full van der Waals contact and high capacitive coupling in the EDL, the top ion-gate devices demonstrated SS less than 150 mV dec^–1^ (Supporting Information Figure 34).

**Figure 4:**
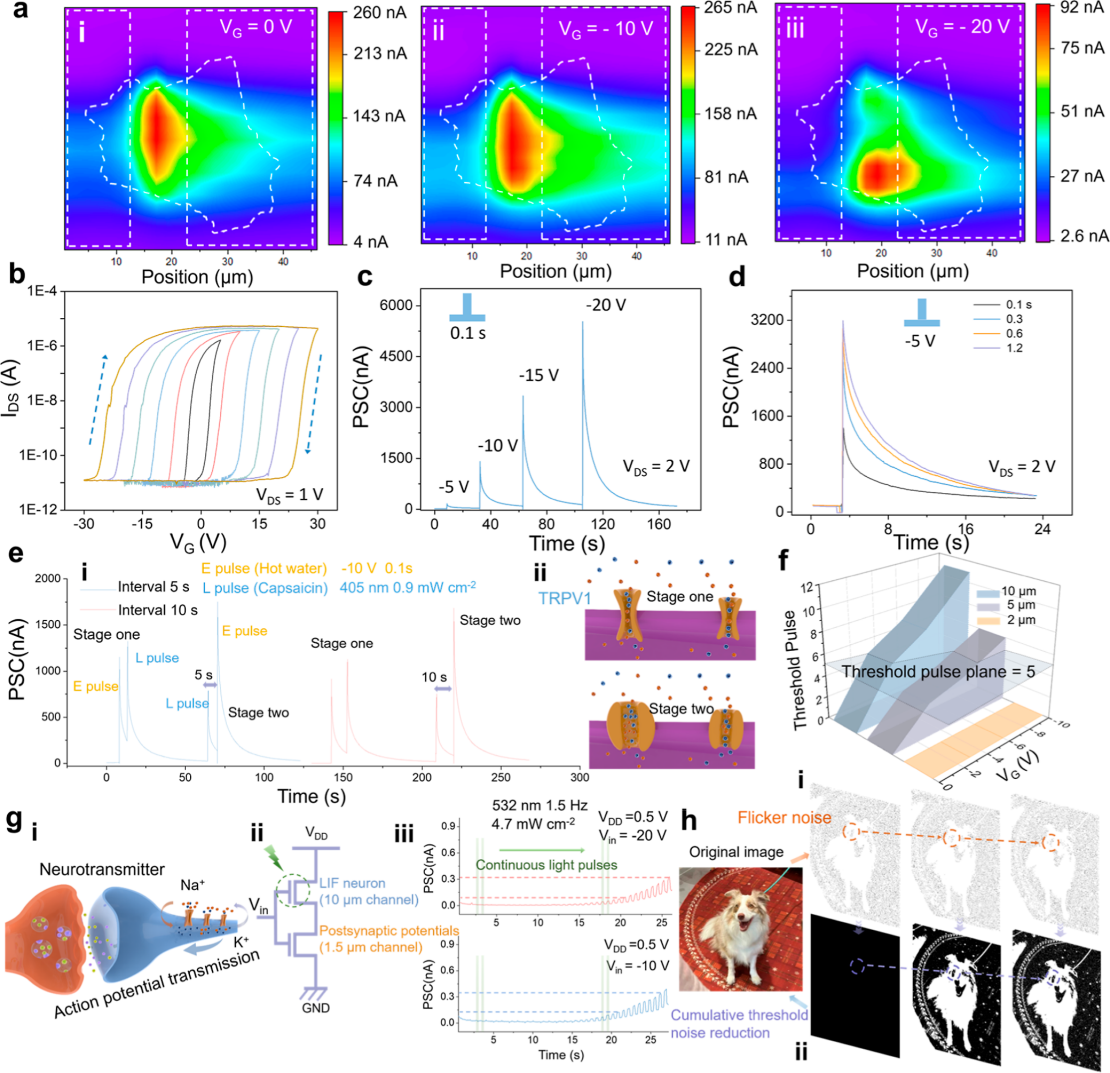
Multimodal response and full-process response. (a) Photocurrent mapping of the FRIV device with 10 μm channel length, under (i) *V*_G_ = 0 V, (ii) *V*_G_ = −10 V, and (iii) *V*_G_ = −20 V. The 520 nm laser source was used, with a power density of 7 mW cm^–2^. (b) Transfer characteristic curves of the FRIV device with different *V*_G_ sweep ranges. The ESPC of the device has (c) different electric pulses and (d) different duration times. (e) (i) Simulation of TRPV1 channel response processes to heat and capsaicin stimulation using electrical and light pulses. E pulse represents −10 V *V*_G_ pulse for 0.1 s, and L pulse represents 405 nm light for 0.5 s (0.9 mW cm^–2^). Extending the interval between the two stimuli from 5 to 10 s still maintains a similar effect. (ii) Ion channel conditions of TRPV1 channels under two stimulation scenarios. (f) Trigger threshold for devices with different channel lengths extracted from Supporting Information Figures 26–29. (g) (i) Schematic diagram of the completed signal transmission process on a nerve through axons and between synapses. (ii) Schematic of a circuit connecting two devices with different channel lengths. (iii) Postsynaptic potentials under different *V*_G_ modulation. The 532 nm light stimulation acted only on the LIF nerve. At the beginning of light stimulation, conductance accumulation in LIF nerves was insufficient, thus preventing the formation of action potentials and the reception of potentiation information at the postsynaptic membrane. (h) Richly detailed image (i) without and (ii) with flicker noise reduction applied based on full-process artificial neurons from (g) (iii).

To realize full-process signal processing, we first extracted trigger conditions for devices with different channel lengths (Figure 4f). This characteristic endows the device with superior noise isolation capabilities (Supporting Information Figure 35). High-frequency white light can be effectively shielded under V_G_ regulation (Supporting Information Movie 1). Then, we connected two devices with channel lengths of 1.5 and 10 μm in series to represent two different transmission processes (Figure 4gi,ii). Sufficient distance between the two devices ensures that the 2 mm diameter laser spot was only directed at one of the devices (Supporting Information Figure 36). Under the regulation of *V*_G_, the front-end LIF neuron was stimulated by light to realize ion transport. Potentials would be accumulated and trigger action potentials under continuous illumination. Then, potentials were transmitted to the presynaptic membrane, enabling neurotransmitter release. Intersynaptic information transfer was subsequently completed, and postsynaptic potentials accumulated. Under different *V*_G_ modulations, the final postsynaptic potential will differ, simulating changes in the sensitivity of different ion channels (Figure 4giii). In most scenes, the presence of high frequencies tends to indicate their exceptionality and importance as the main subjects. In the case of flicker noise, unnatural random variations in brightness in space and time can alter the frequency randomness, affecting the prominence of the subject’s features (Figure 4hi). When we used the threshold response (Figure 4giii) of FRIV neurons, this system, built from sensing and threshold-filtering units, automatically filtered out the flicker. The subject accumulates current in the time domain and breaks the threshold through frequent stimulation (Figure 4hii). Many details are shown and preserved compared with the original image. In contrast, flickering random variations make it difficult to accumulate detail in the image. Supporting Information Movies 2 and 3 show the changes in the image with and without flicker noise reduction, respectively.

Being able to quickly and effectively recognize moving objects, even with flicker noise suppression, is an important foundation for realizing the next generation of artificial vision applications. Figure 3e depicts the photoresponse elicited by the FRIV device when exposed to a series of 32, 5 bit continuous optical pulses, ranging from 00000 to 11111. These 32 sequences of optical pulses generate 32 different states, underscoring the FRIV device’s powerful ability to transduce intricate spatiotemporal signals into reservoir states.^45^ This quality substantiates its prospective utility in in-sensor memory computing. When the FRIV device is assembled into a pixel array, we suggest using the in-sensor reservoir for motion recognition, as demonstrated in Figure 5a–c. The reservoir can transpose complex inputs into reservoir states, which are then used for feature extraction. Subsequently, these extracted features are used by the linear output layer for classification. RC possesses the dissimilar advantage of requiring minimal training. More specifically, the input and reservoir layers remain static, while only the output layer weights are subject to training. This characteristic renders RC particularly well-suited to edge learning applications.^46^

**Figure 5:**
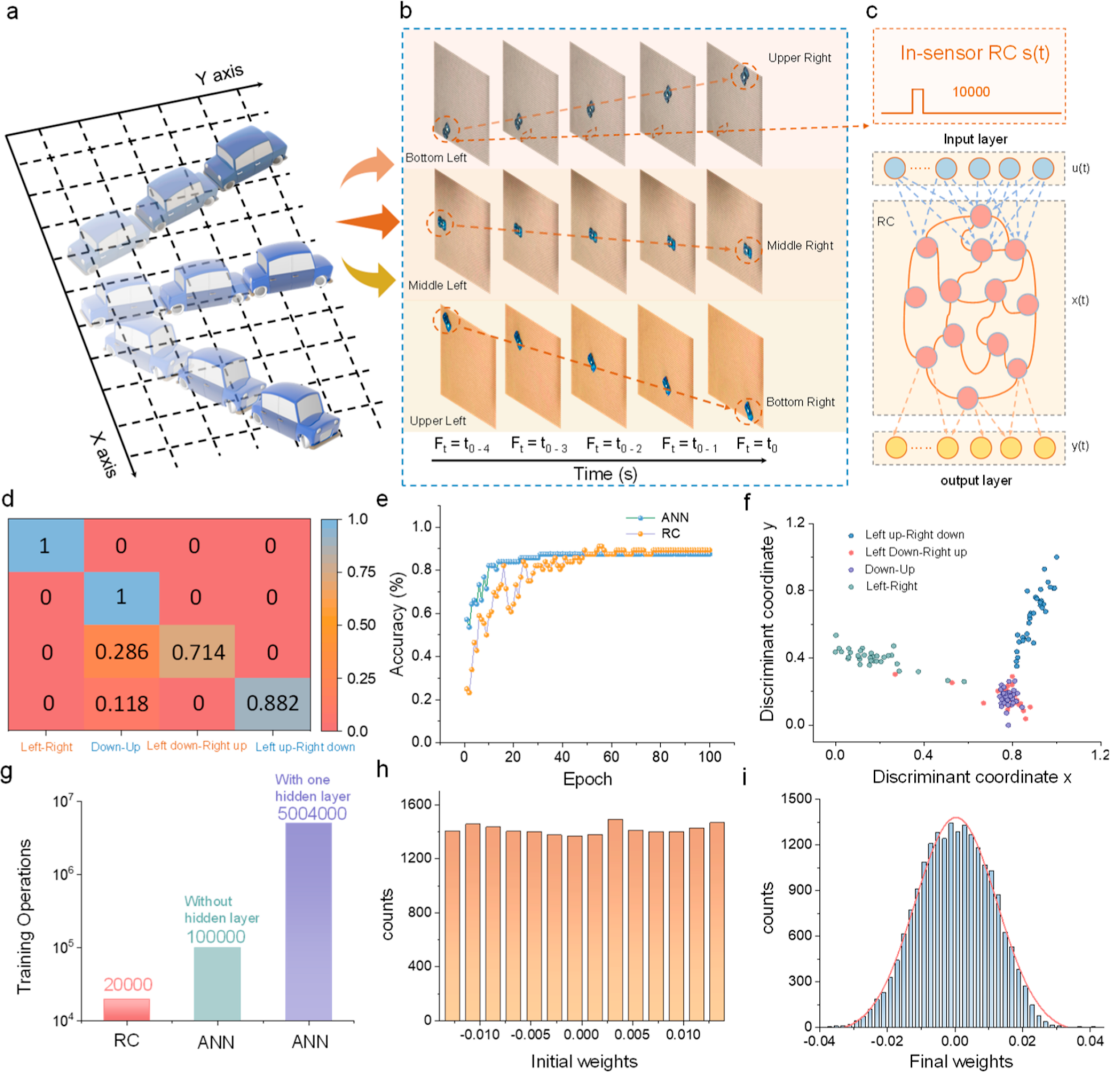
Dynamic perception with FRIV-based RC system. (a) Schematic diagram of the trajectory of the car. (b) The trajectories of the car in different directions and the five consecutive frames. (c) Schematic illustration of the RC system for classifying the car motion. (d) Confusion matrix for classifying various cart trajectories. (e) The performance of the FRIV-based RC system compared with that of the software baseline, which employs a one-layer fully connected neural network. (f) Two-dimensional clustering of feature vectors for dimensionality reduction using LDA. (g) Number of training operations for RC and different ANN networks. The FRIV-based RC system showed obvious advantages in energy consumption and operational simplicity. Different conductance distributions of (h) initial state and (i) after training.

The in-sensor reservoir records vehicular motion trajectories using a tailored data set composed of four emblematic classes: ″from left to right″, ″from bottom to top″, ″from bottom left to top right″, and ″from bottom right to top left″, as illustrated in Figure 5a. Supporting Information Movie 4 demonstrates the car’s trajectory at 240 FPS (frames per second). To encapsulate spatial and temporal data from successive frames into compact representations, each movement was segmented into five consecutive frames (Ft4, Ft3, Ft2, Ft1, and Ft0). These frames correspond to five different instances during vehicular motion, as depicted in Figure 5b. The temporal progression of each pixel was condensed into a 5 bit sequence, s­(t), comprising 32 potential binary vectors, each encoded as a light intensity.^47^ The FRIV device properties of the pixel array enable individual transistors to generate features, specifically conductance, corresponding to 1 of 32 different illumination patterns, as shown in Figure 3e. This approach embeds temporal pixel evolutions in the conductance of FRIV devices. These conductance values were concatenated to capture the temporal feature of the motion, thereby providing an integrated and compact representation of the spatiotemporal data.

The readout map, depicted as a fully connected layer in this context, served as a trainable classification head for the features extracted by the FRIV device. Figure 5d presents the confusion matrix for classifying diverse vehicular movements. The prevalence of dominant diagonal elements indicates a high accuracy within each category. The results of our training were documented in Figure 5e, revealing an overall simulated accuracy rate of 89%. This is based on the behavior of FRIV devices, represented by orange dots, and aligns with the performance of the software baseline, which employs a one-layer fully connected neural network (indicated by blue dots). Moreover, we provide two-dimensional clustering of feature vectors extracted from FRIV devices in Figure 5f, utilizing linear discriminant analysis (LDA) for dimensionality reduction. Then, Figure 5g compares the training parameters required for various configurations, including an RC system, a single-layer fully connected neural network, and a two-layer fully connected neural network. It is observed that the number of parameters required for a deep, fully connected network escalates rapidly, while this figure remains constant for the RC system. This observation underscores the benefits of RC for cost-effective real-time edge learning given its capacity to maintain manageable complexity even as depth increases. Lastly, Figure 5h,i illustrate the initial and final weight distributions throughout the readout layer. Notably, these features undergo efficient nonlinear transformations due to short-term memory effects.

## Conclusion

In summary, we successfully developed a Tr-COF/MoS_2_ heterojunction with a suspended-channel architecture to replicate the complete nerve signal transmission process under gate-voltage modulation. This design effectively separates photogenerated carriers at the heterojunction interface via energy-band effects. Photogenerated holes are trapped by the porous laminated Tr-COF, creating a photogate effect that extends the relaxation time for the channel carriers. Owing to their superior optoelectronic capabilities, artificial neurons facilitate a neuromorphic response to high-frequency visible-light signals and achieve an impressive information transmission rate of 2100 bits s^–1^. Gate-voltage regulation further introduces a transport barrier via the suspended S/D junction, mimicking action potential accumulation and firing behavior in leaky integrate-and-fire neurons. Importantly, the synapse-neuron-coupled architecture intrinsically suppresses flicker noise at the sensing front end through temporal accumulation and threshold-based filtering, rather than relying on backend digital deflickering algorithms. This in-sensor computing mechanism enhances detail preservation while reducing data movement, latency, and energy consumption, highlighting a system-level advantage over conventional vision architectures. Building on these device-level neuromorphic dynamics, we demonstrate a proof-of-concept trajectory-recognition task using in-sensor reservoir computing to establish a clear link between physical temporal responses and system-level motion perception. The reported classification results are not intended to represent the upper bound of motion complexity but rather to validate the capability to encode and process motion-relevant spatiotemporal information directly at the sensor level. Owing to its continuous-time dynamics and array-level scalability, the proposed platform provides an interpretable and extensible hardware foundation for future low-power, real-time neuromorphic motion-perception systems.

## Methods

### Material Transfer and Device Preparation

The Au S/D electrodes (50 nm) were patterned by photolithography with different channel lengths and then deposited on 270 nm-thick SiO_2_/Si substrates by thermal evaporation. The MoS_2_ nanoflake was mechanically exfoliated onto a poly­(dimethylsiloxane) (PDMS) substrate and then transferred accurately onto the S/D channel. The triazine-based COF was obtained using the aggregation of 4,4′4″-(1,3,5-triazine-2,4,6-triyl) trianiline and ace-naphthenequinone monomers. The 10 mg Tr-COF powder was dispersed in 1 mL of ethanol solvent and ultrasonicated for 20 min. Then, the spin-coating method was used to fabricate a Tr-COF/MoS_2_ heterojunction at 2000 rpm for 60 s. For ion-gate fabrication, the poly­(vinylidene difluoride-*co*-hexafluoropropylene) [P­(VDF-HFP)], 1-ethyl-3-methylimidazolium bis (trifluoromethylsulfonyl) imide [(EMIM)­(TFSI)], and acetone with a 1:4:7 mass ratio were mixed. Then, the solution was heated and stirred for over 6 h until completely dissolved. The spin-coating process was adopted to fabricate an ion-gate film on 270 nm-thick SiO_2_/Si substrates at 500 rpm for 20 s, followed by annealing at 70 °C for 2 h. Then, the ion gate was physically transferred to the multigate device channel to serve as the top gate.

### Measurement and Characterization

The optoelectronic performance of the FETs was then characterized with a standard electrical probe station and an Agilent 4155C semiconductor analyzer (Agilent Technologies, Santa Clara, CA). The morphologies and EDS mapping of Tr-COF were examined by using scanning electron microscopy (SEM, Quanta 450 FEG, FEI) and high-resolution transmission electron microscopy (HRTEM, Thermo Scientific, Talos F200X). The morphologies of MoS_2_ were confirmed by AFM (Bruker Dimension Icon AFM). Raman and PL spectroscopy were used to characterize the quality and band gap of MoS_2_ (FLS980). The power of the light stimuli was calibrated and measured by using a power meter (PM400, Thorlabs). The irradiation power was tuned by a modulator (AFG 2005, Good Will) connected to the laser source. The crystallinity and microstructure of Tr-COF were confirmed by XRD (D2 Phaser with Cu Kα radiation, Bruker) and FTIR (Thermo Fisher Nicolet iS5 system).

## Supplementary Material











## Notes

Video showing high-frequency white light can be effectively shielded under V_G_ regulation (MP4)
Changes in the image with flicker noise reduction (MP4)
Mathematical explanations of transmission efficiency; and additional experimental details, including materials characterization and performance and efficacy characterization (PDF)
Changes in the image without flicker noise reduction (MP4)
The car’s trajectory at 240 FPS (MP4)
