# Supplementary material for: Flicker-Suppressed Neuromorphic Unit for Dynamic Vision Processing
Source: ACS Nano. 2026 Feb 25;20(9):7640–51. doi: 10.1021/acsnano.5c18939 (PMC12981026; doi:10.1021/acsnano.5c18939)
Supplement: Supplementary file 3 [file nn5c18939_si_003.pdf]

Supplementary Information

Flicker-Suppressed Neuromorphic Unit for Dynamic Vision Processing

Pengshan Xie[1,#], Shuhui Shi[2,#], Lei Ran[3,#], Chunhua Wang[3,*], Dengji Li[1], Yuxuan Zhang[1], Yiyang Wei[4], Quan Quan[1], Bowen Li[1,5], You Meng[1,6], Weijun Wang[1], Boxiang Gao[1], Changyong Lan[4], Michael K.H. Leung[3,*], Zhongrui Wang[2,*], Johnny C. Ho[1,5,6,7,*]

1 Department of Materials Science and Engineering, City University of Hong Kong, Kowloon 999077, Hong Kong SAR.

2 School of Microelectronics, Southern University of Science and Technology, Shenzhen 518055, China.

3 School of Energy and Environment, City University of Hong Kong, Kowloon 999077, Hong Kong SAR.

4 School of Optoelectronic Science and Engineering, University of Electronic Science and Technology of China, Chengdu, 611731, China.

5 Shenzhen Research Institute, City University of Hong Kong, Shenzhen 518057, China

6 State Key Laboratory of Terahertz and Millimeter Waves, City University of Hong Kong, Kowloon 999077, Hong Kong SAR.

7 Institute for Materials Chemistry and Engineering, Kyushu University, Fukuoka 816-8580, Japan.

# These authors contributed equally: Pengshan Xie, Shuhui Shi, Lei Ran

* Corresponding authors E-mail: johnnyho@cityu.edu.hk (J. C. Ho), wangzr@sustech.edu.cn (Z. Wang), mkh.leung@cityu.edu.hk (M. K. H. Leung), chunhuaw@umich.edu (C. Wang).

Table of Contents

1. Supplementary Note I……………………………..............................……….2

2. Supplementary Note I……………………………....…………………………..2

3. Supporting Figures………………………………………………………........… 2

4. Reference……………………………………………….…..……………….......…..15

1. Supplementary Note I

The Fourier transform is a mathematical tool for converting a time-domain signal into a frequency-domain representation. It reveals the frequency components of a signal by decomposing it into sine and cosine waves of different frequencies.[1] Specifically:

$$S(f) = \int_{-\infty}^{\infty} S(t) * e^{-2\pi itf}\, dt \quad (1)$$

$$N(f) = \int_{-\infty}^{\infty} N(t) * e^{-2\pi itf}\, dt \quad (2)$$

where S(t) is the time-domain photocurrent response of the device, and N(t) is the time-domain dark current response of the device.

The signal-to-noise ratio (SNR) is widely used to compare the strength of a desired signal to that of background noise. The frequency domain SNR (f) is the ratio of the signal power spectrum ($|S(f)|^2$) to the noise power spectrum ($|N(f)|^2$) at a specific frequency, given by:

$$SNR(f) = \frac{|S(f)|2}{|N(f)|2} \quad (3)$$

2. Supplementary Note II

In order to assess the information transmission capacity of neurons, the information transmission rate (r) is an effective metric parameter, shown as:

$$r = \int_{1}^{100} df\, log_2[1 + SNR(f)] \quad (4)$$

where the upper and lower limits of the integral correspond to the frequency domain range of the SNR. r visually describes the amount of information transmitted per unit of time (bits second^{-1}).[2]

3. Supporting Figures

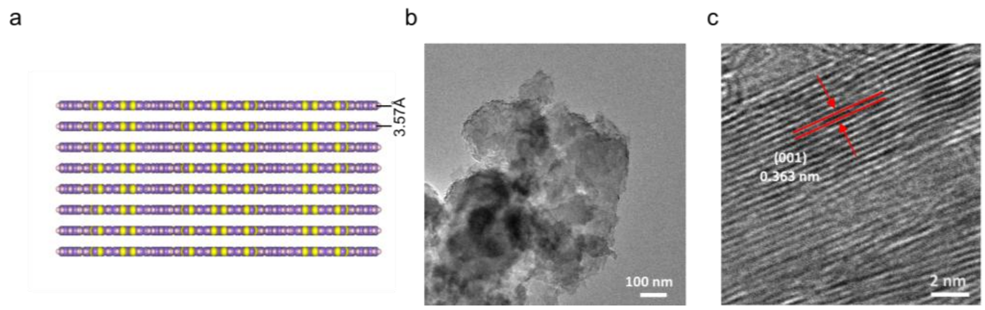

Supplementary Fig. 1| a, Schematic diagram of the laminar structure of Tr-COF. The **b**, TEM image, and **c**, HRTEM image of Tr-COF.

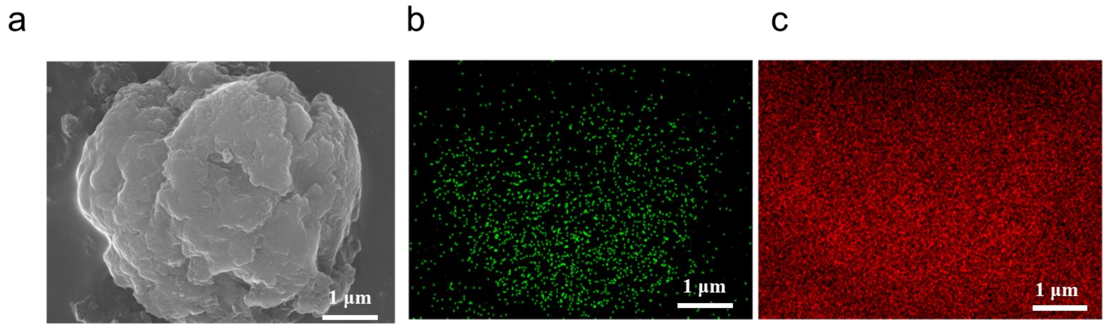

Supplementary Fig. 2| a, SEM image of the Tr-COF. The EDS mapping of **b**, C, and **c**, N elements.

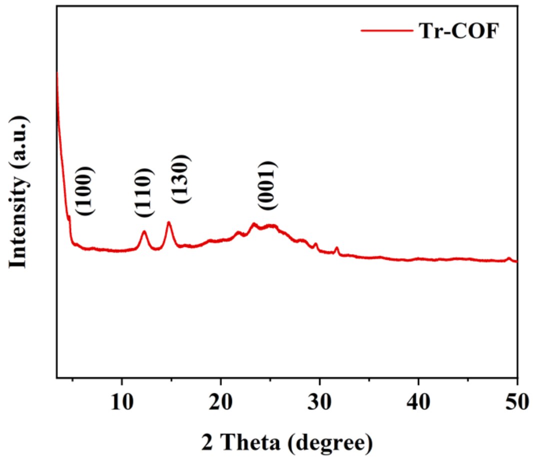

Supplementary Fig. 3| The powder X-ray diffraction pattern of the Tr-COF.

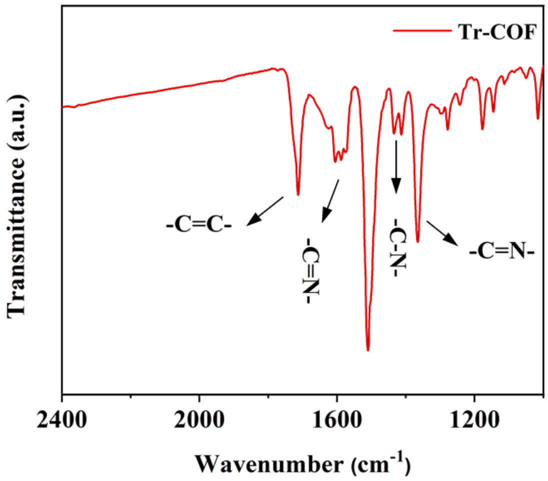

Supplementary Fig. 4| The Fourier transform infrared spectroscopy (FTIR) spectra of the Tr-COF.

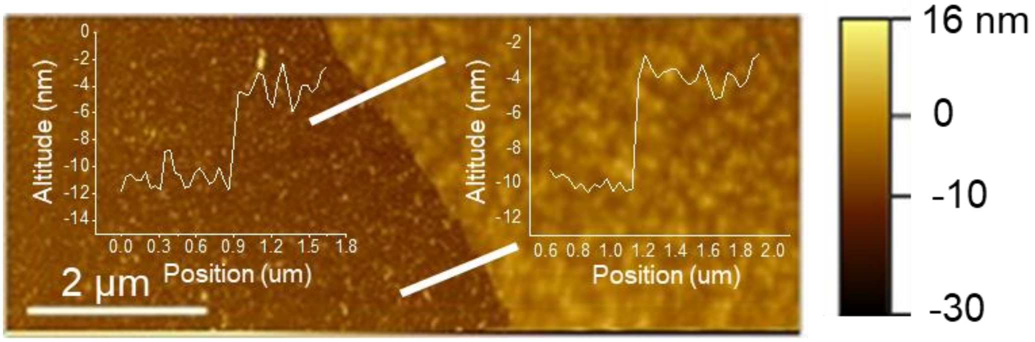

Supplementary Fig. 5| The AFM image for the thickness of mechanical exfoliation of MoS_2.

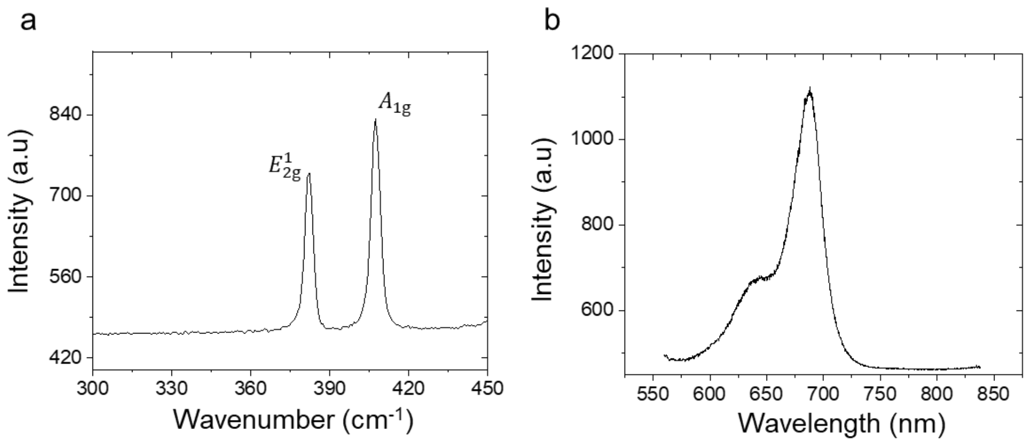

Supplementary Fig. 6| a, Raman spectrum of the few-layer MoS_2. **b**, The photoluminescence spectrum (PL) of the few-layer MoS_2 to confirm the band gap.

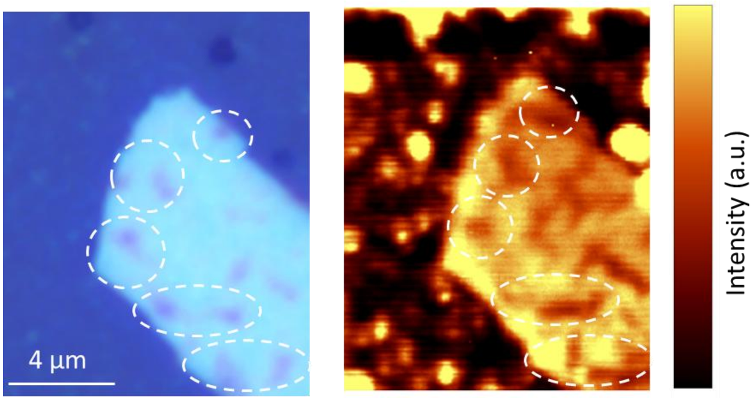

Supplementary Fig. 7| The PL mapping of the MoS_2/Tr-COF interface.

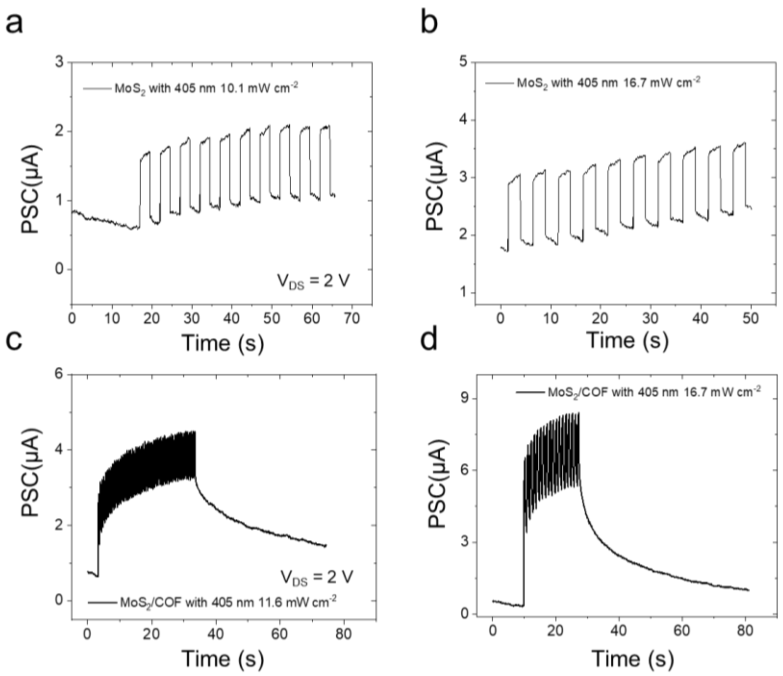

Supplementary Fig. 8| The EPSC of the device with only MoS_2 under 405 nm light **a**, 10.1 mW cm^{-2}, **b**, 16.7 mW cm^{-2}. The EPSC of the device with MoS_2/Tr-COF heterojunction under 405 nm light **c**, 11.6 mW cm^{-2}, **d**, 16.7 mW cm^{-2}.

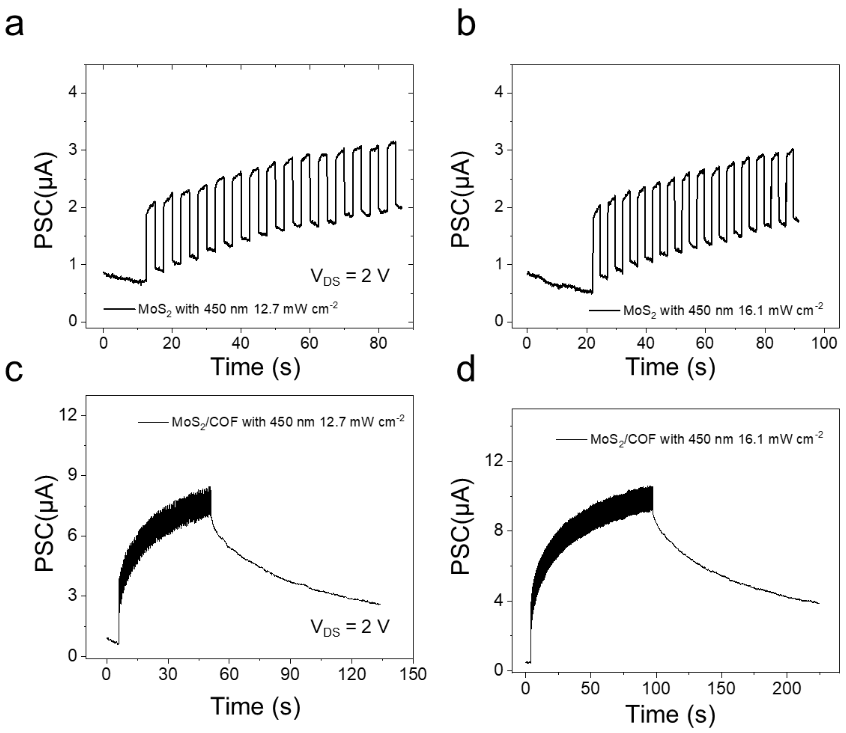

Supplementary Fig. 9| The EPSC of the device with only MoS_2 under 450 nm light **a**, 12.7 mW cm^{-2}, **b**, 16.1 mW cm^{-2}. The EPSC of the device with MoS_2/Tr-COF heterojunction under 450 nm light **c**, 12.7 mW cm^{-2}, **d**, 16.1 mW cm^{-2}.

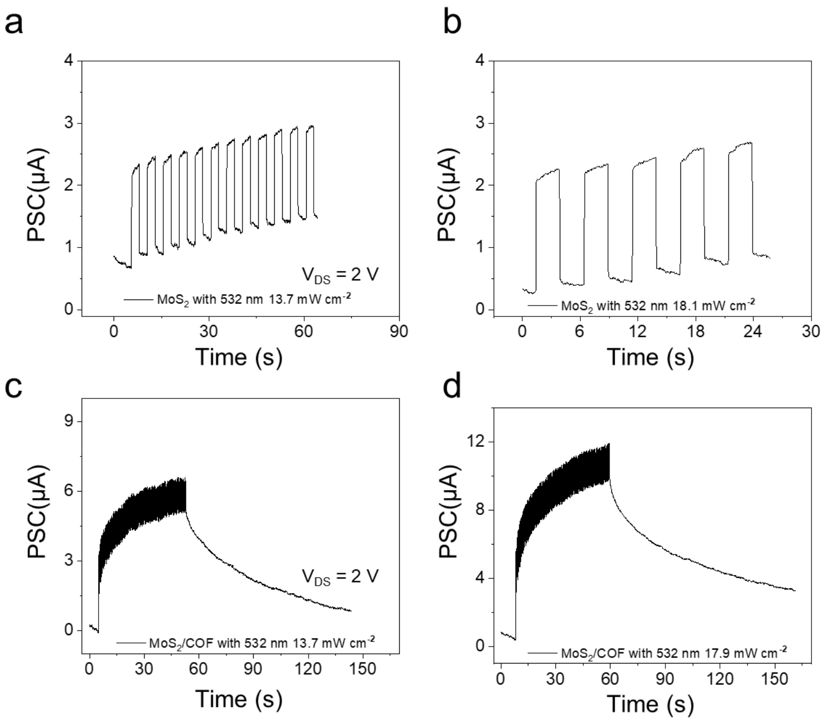

Supplementary Fig. 10| The EPSC of the device with only MoS_2 under 532 nm light **a**, 13.7 mW cm^{-2}, **b**, 18.1 mW cm^{-2}. The EPSC of the device with MoS_2/Tr-COF heterojunction under 532 nm light **c**, 13.7 mW cm^{-2}, **d**, 17.9 mW cm^{-2}.

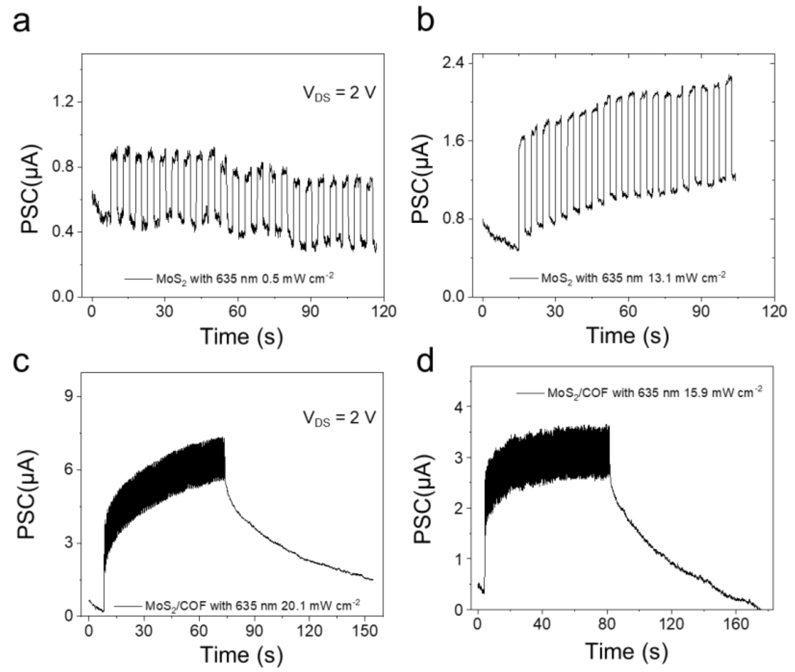

Supplementary Fig. 11| The EPSC of the device with only MoS_2 under 635 nm light **a**, 0.5 mW cm^{-2}, **b**, 13.1 mW cm^{-2}. The EPSC of the device with MoS_2/Tr-COF heterojunction under 635 nm light **c**, 20.1 mW cm^{-2}, **d**, 15.9 mW cm^{-2}.

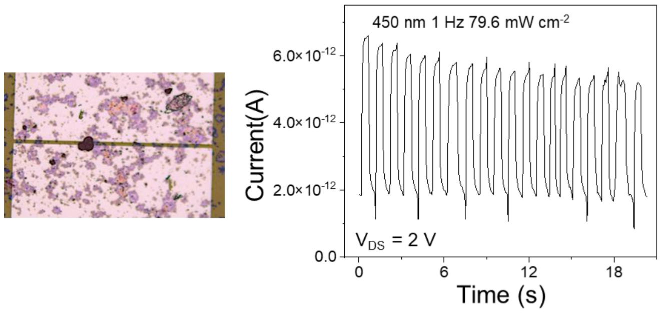

Supplementary Fig. 12| a, Photographs of Tr-COF devices (2 μm channel). **b**, Corresponding photoresponse in the blue light range.

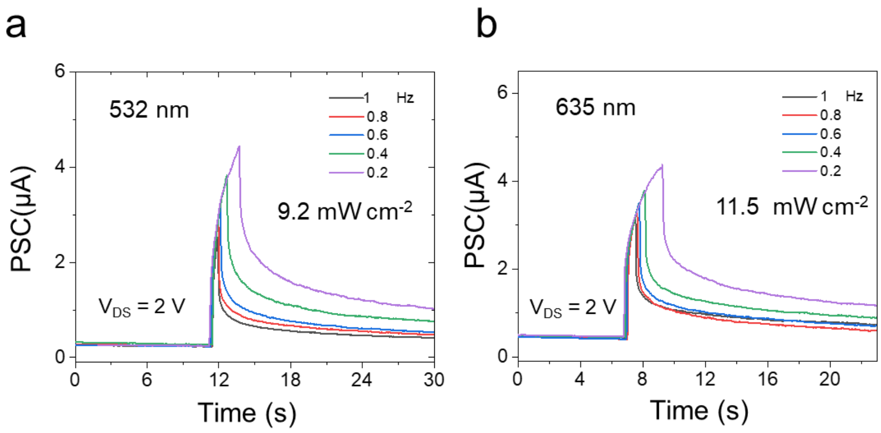

Supplementary Fig. 13| The SDDP characteristics of the device under a, 532 nm and b, 635 nm wavelength light with different irradiation times.

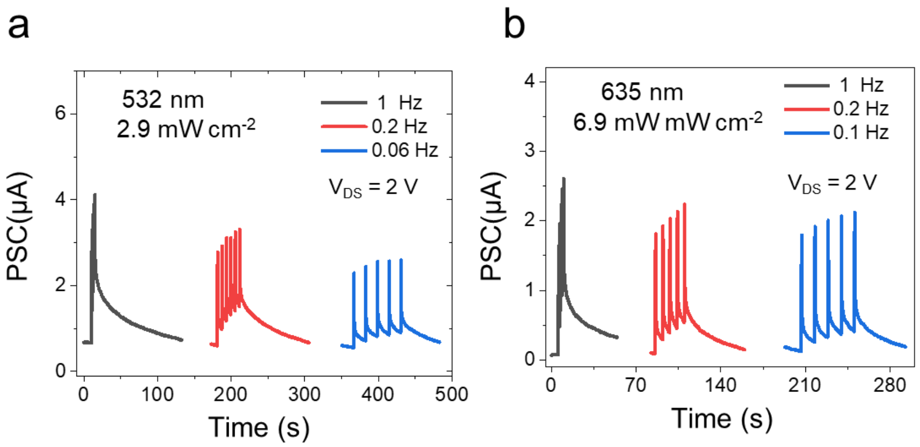

Supplementary Fig. 14| The SFDP characteristics of the device with **a**, 532 nm and **b**, 635 nm light.

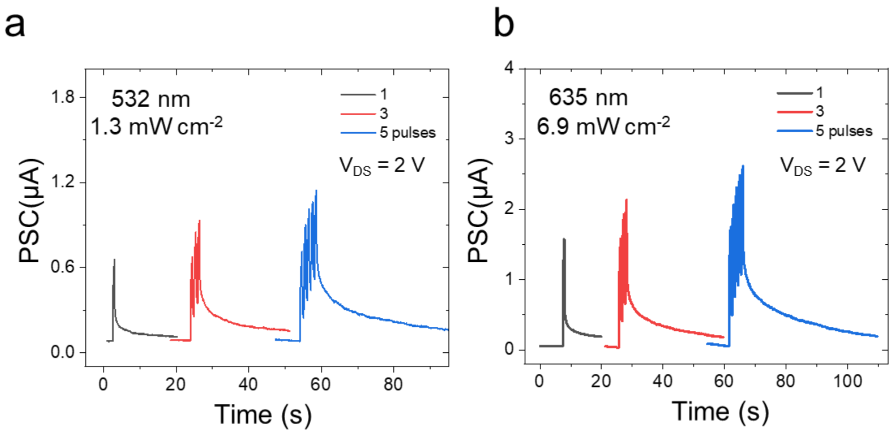

Supplementary Fig. 15| The SNDP characteristics of the device with **a**, 532 nm and **b**, 635 nm light.

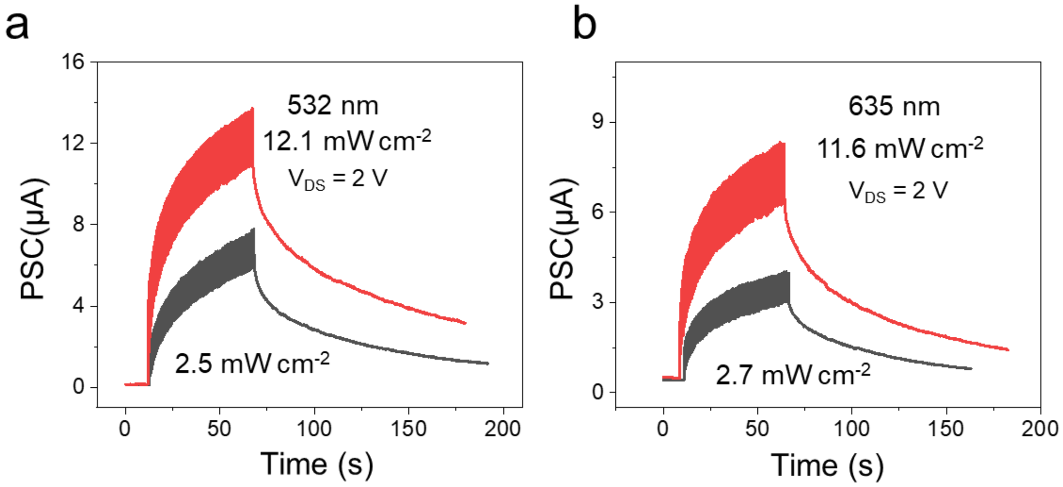

Supplementary Fig. 16| The LTP characteristics of the device with 100 continuous stimuli with **a**, 532 nm and **b**, 635 nm light.

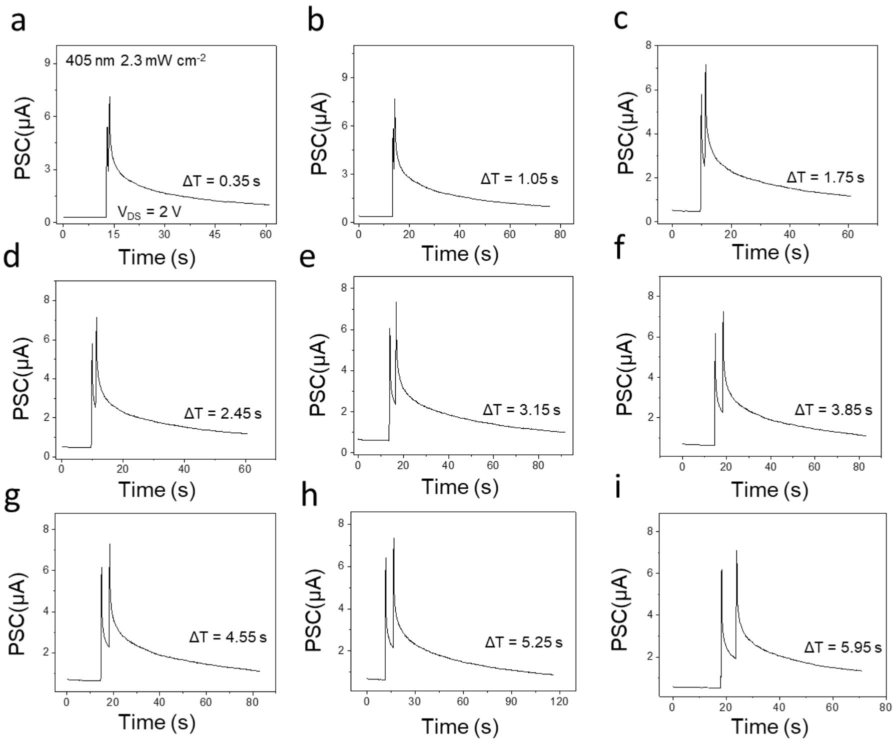

Supplementary Fig. 17| The PPF characteristics of the device under 405 nm light. The interval of two pulses was changed to **a**, 0.35 s, **b**, 1.05 s, **c**, 1.75 s, **d**, 2.45 s, **e**, 3.15 s, **f**, 3.85 s, **g**, 4.55 s, **h**, 5.25 s and **i**, 5.95 s.

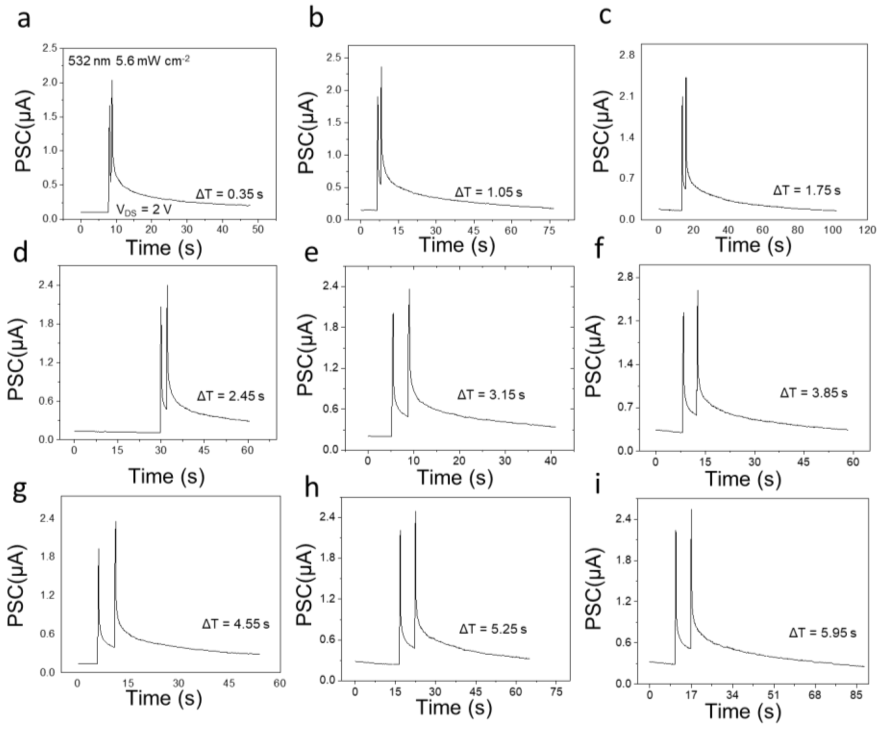

Supplementary Fig. 18| The PPF characteristics of the device under 532 nm light. The interval of two pulses was changed to **a**, 0.35 s, **b**, 1.05 s, **c**, 1.75 s, **d**, 2.45 s, **e**, 3.15 s, **f**, 3.85 s, **g**, 4.55 s, **h**, 5.25 s and **i**, 5.95 s.

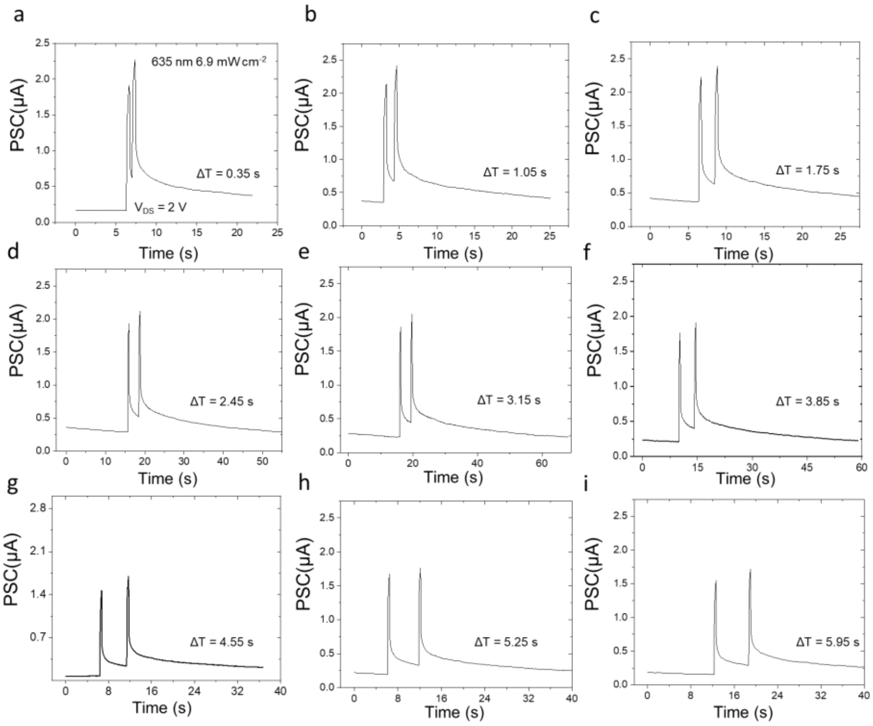

Supplementary Fig. 19| The PPF characteristics of the device under 635 nm light. The interval of two pulses was changed to **a**, 0.35 s, **b**, 1.05 s, **c**, 1.75 s, **d**, 2.45 s, **e**, 3.15 s, **f**, 3.85 s, **g**, 4.55 s, **h**, 5.25 s and **i**, 5.95 s.

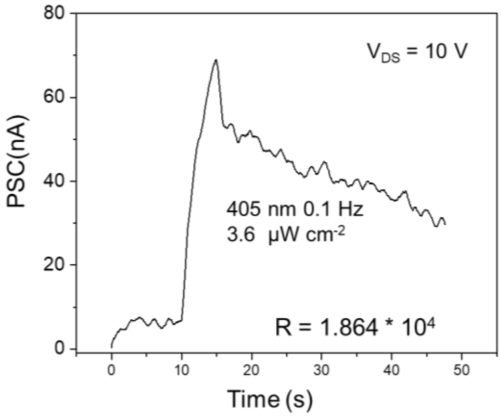

Supplementary Fig. 20| Photoresponse of the device with single 405 nm light (5s, 3.6 μW cm^{-2}). The responsivity (R) was obtained by the equation $R = \frac{I_{ph}}{PS}$, where I_{ph} ($I_{ph} = I_{light} - I_{dark}$), P and S are the photocurrent, power density, and effective illumination area, respectively. Here, I_{light} is the photocurrent after irradiation with light.[3]

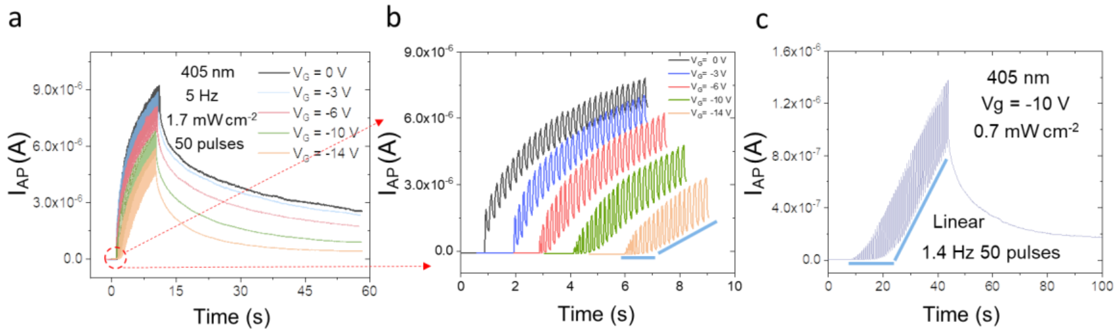

Supplementary Fig. 21| a, The LIF neuron response with different V_G modulation under 405 nm continuous light inputs. **b**, the details of the initial response. **c**, The Linear response after excitation of the LIF neuron with 405 nm light.

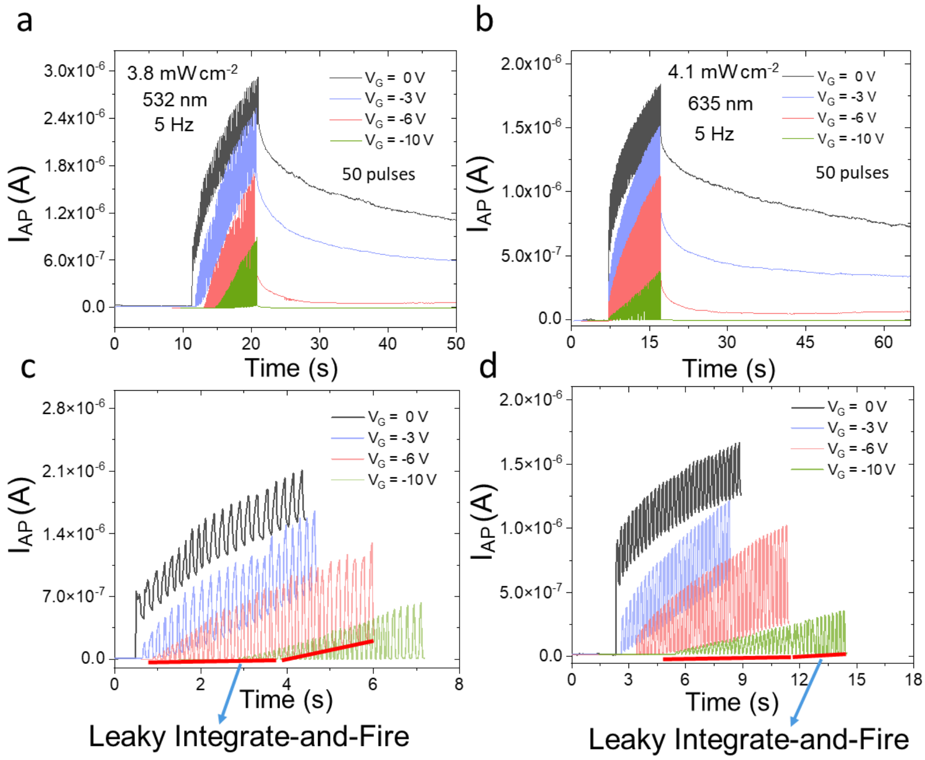

Supplementary Fig. 22| The LIF neuron response with different V_G modulation under **a**, 532 nm, and **b**, 635 nm continuous light inputs. The details of the initial response under **c**, 532 nm, and **d**, 635 nm light irradiation.

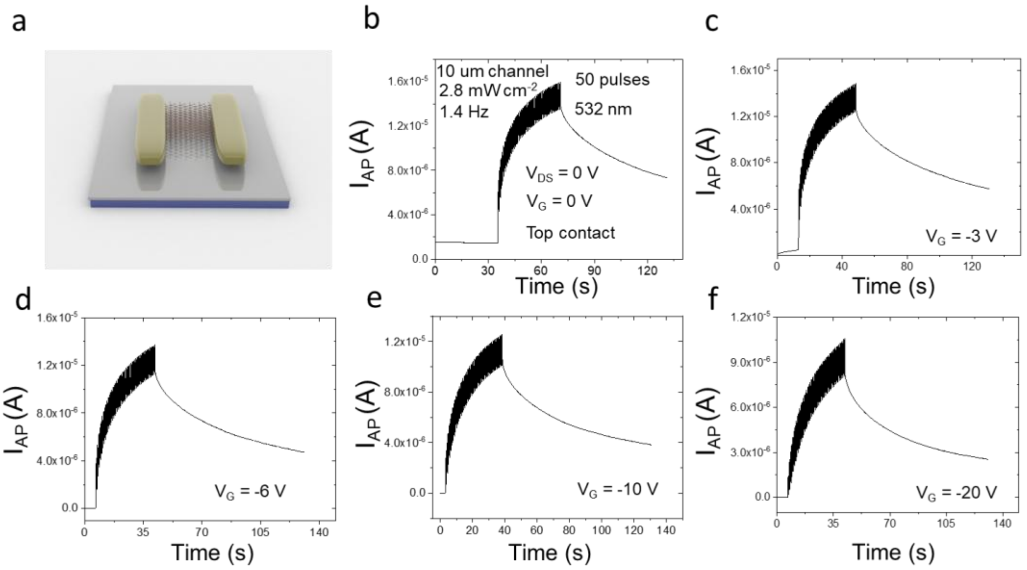

Supplementary Fig. 23| a, Schematic diagram of the bottom-gate top-contact device structure. The photoresponse of the device with a 10 μm-long channel and 532 nm light input under **b**, V_G = 0, **c**, V_G = -3, **d**, V_G = -6, **e**, V_G = -10, and **f**, V_G = -20.

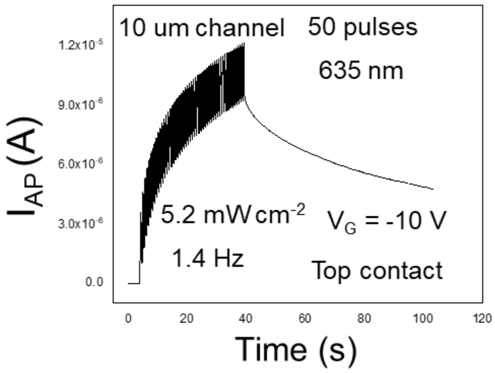

Supplementary Fig. 24| The photoresponse of the device with 635 nm light inputs under V_G = -10 V.

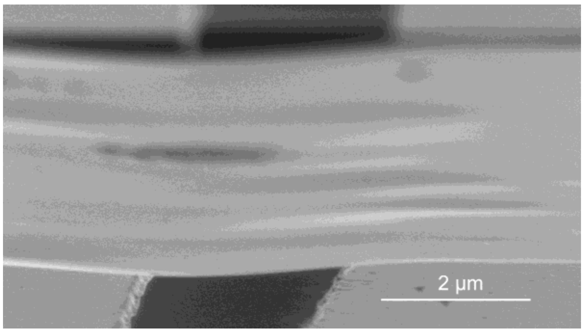

Supplementary Fig. 25| The SEM image of a 2 μm long MoS_2 channel.

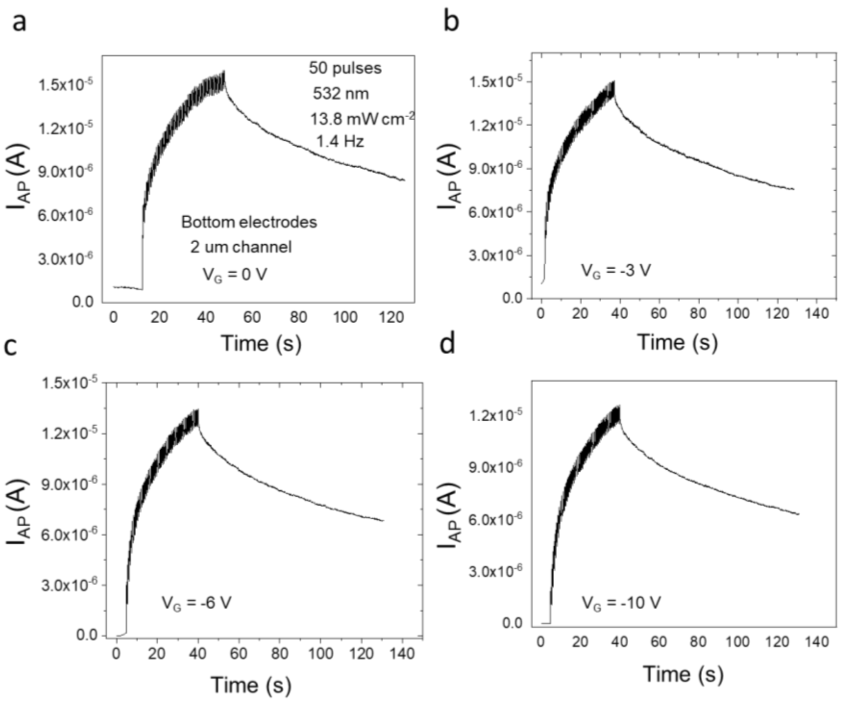

Supplementary Fig. 26| The photoresponse of a bottom-gate bottom-contact device with a 2 μm long channel and 532 nm light pulses under **a**, V_G = 0, **b**, V_G = -3, **c**, V_G = -6, **d**, V_G = -10.

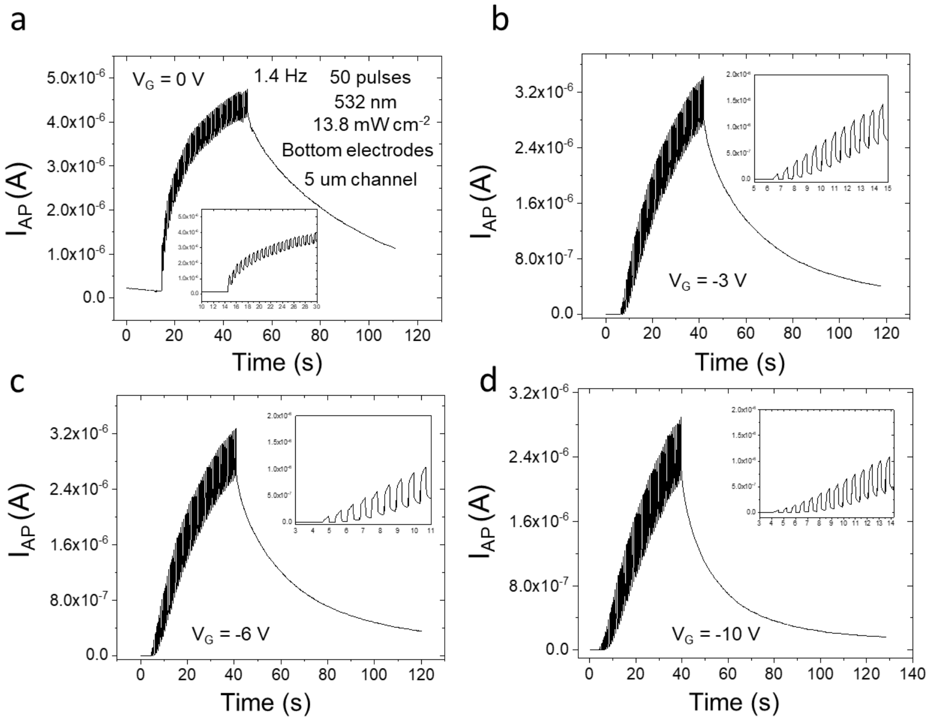

Supplementary Fig. 27| The photoresponse of a bottom-gate bottom-contact device with a 5 μm long channel and 532 nm light pulses under **a**, V_G = 0, **b**, V_G = -3, **c**, V_G = -6, **d**, V_G = -10.

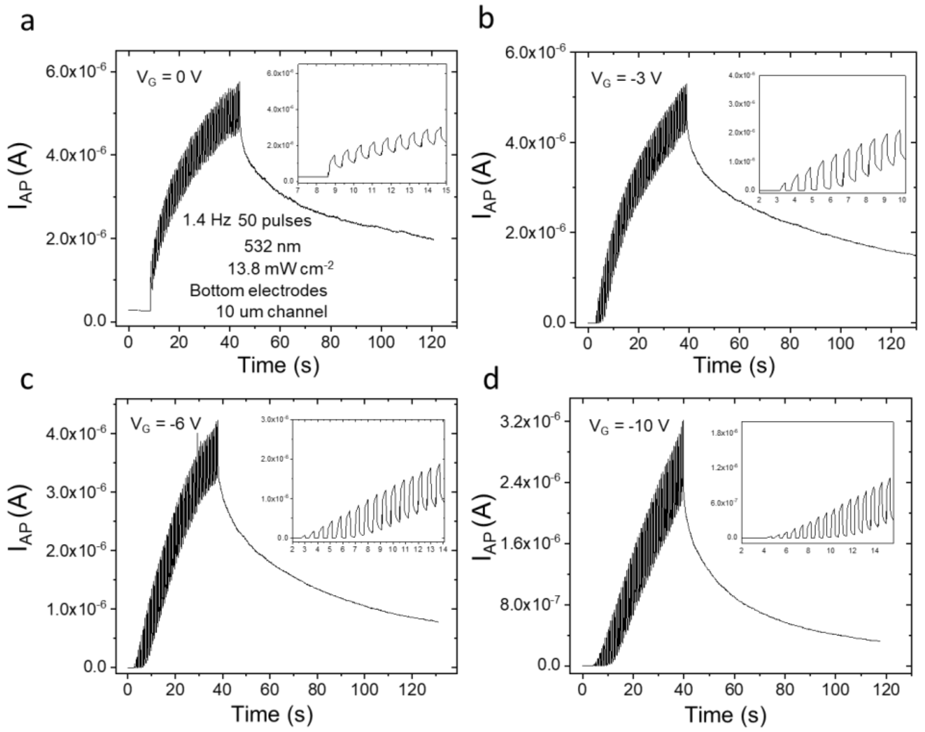

Supplementary Fig. 28| The photoresponse of a bottom-gate bottom-contact device with a 10 μm long channel and 532 nm light pulses under **a**, V_G = 0, **b**, V_G = -3, **c**, V_G = -6, **d**, V_G = -10.

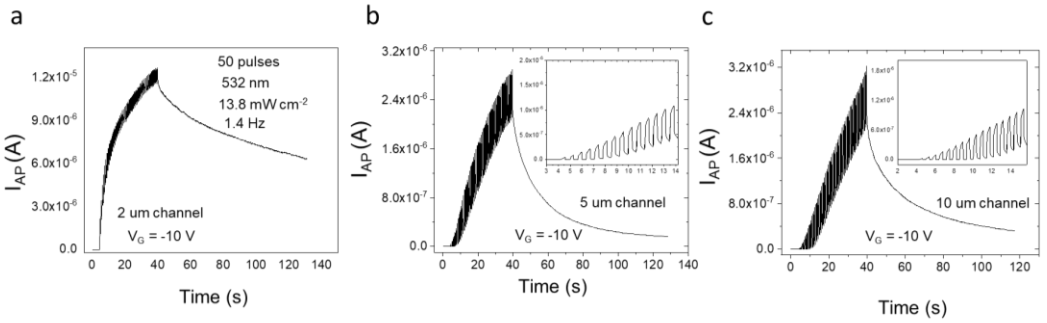

Supplementary Fig. 29| The photoresponse of devices under the same test conditions with **a**, 2 μm long channel, **b**, 5 μm long channel, and **c**, 10 μm long channel.

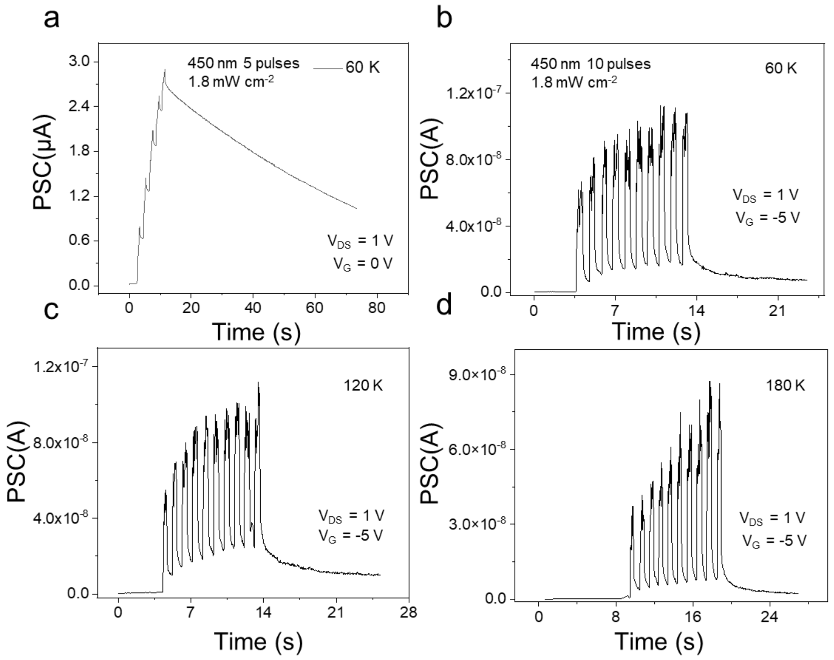

Supplementary Fig. 30| a, The EPSC of the 5 μm channel device under 60 K testing conditions. Accumulation of photoconductivity modulated by V_G under varying temperature conditions. **b**, 60 K. **c**, 120 K. **d**, 180 K.

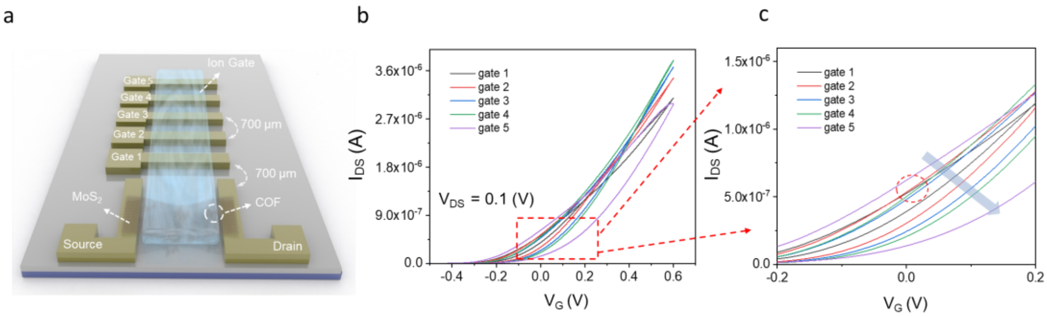

Supplementary Fig. 31| a, Schematic diagram of multi-top ion gate device structure. **b**, Transfer characteristic curves for different gate modulations, and **c**, the details of the hysteresis window.

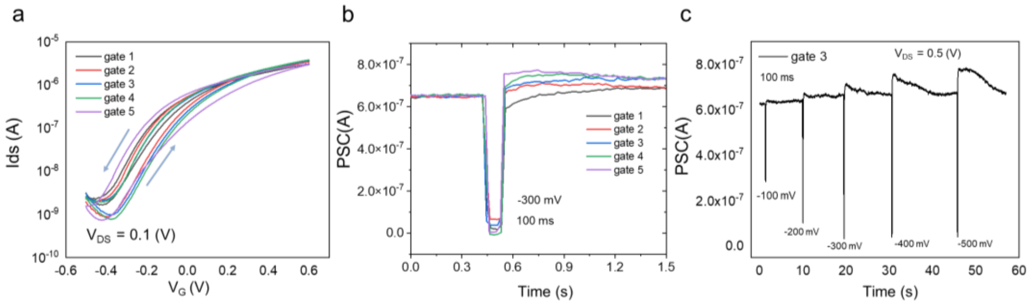

Supplementary Fig. 32| a, Transfer characteristic curves in log coordinates. **b**, The EPSC of the electric pulse applied to different ion gates. **c**, The EPSC induced by the ion gate 3.

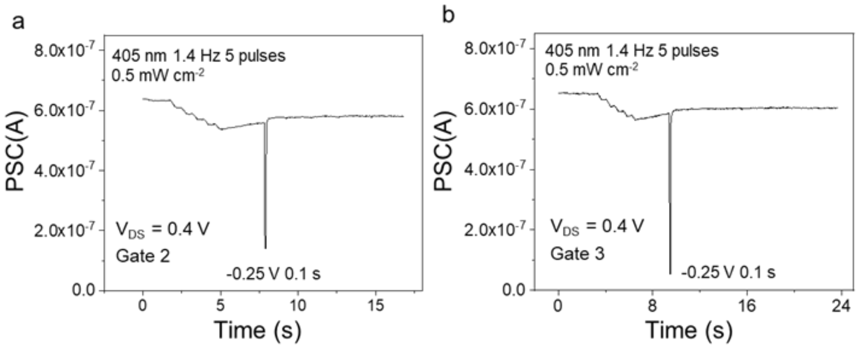

Supplementary Fig. 33| Optoelectronic synergistic regulation with **a**, gate 2, and **b**, gate 3. This negative photoconductivity can be attributed to the photo-induced electrochemical activity of the Tr-COF under electrolyte conditions. Specifically, upon illumination, the Tr-COF layer participates in reduction reactions that rapidly consume the photogenerated electrons. As a result, these electrons are effectively trapped or depleted and are unable to transfer into the MoS_2 channel. Meanwhile, driven by the interfacial band alignment, photogenerated holes in MoS_2 are transferred into the Tr-COF, leading to the accumulation of positive charges in the Tr-COF layer. This enhanced positive charge density at the interface acts as a scattering and electrostatic perturbation center, increasing carrier scattering in the MoS_2 channel.

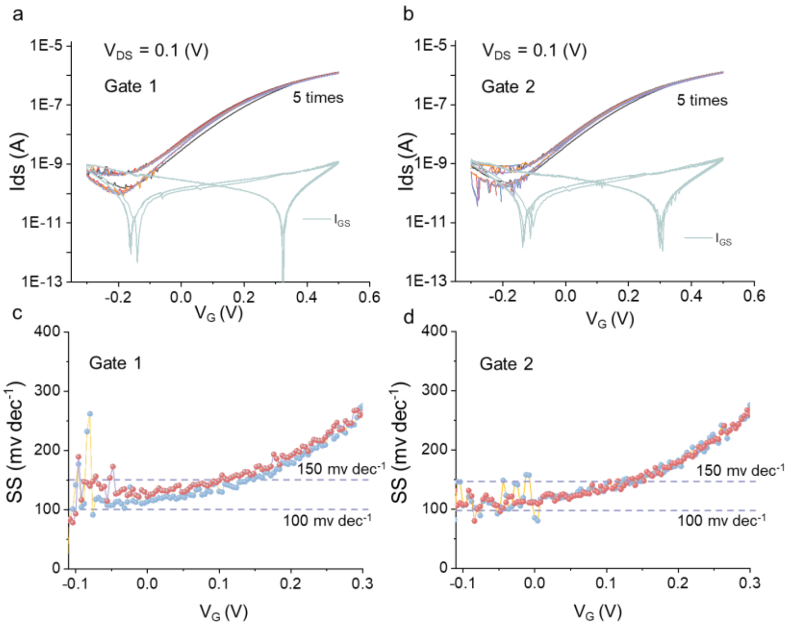

Supplementary Fig. 34| Transfer characteristic curves of **a**, ion gate 1, and **b**, ion gate 2. The SS as a function of V_G with **c**, ion gate 1, and **d**, ion gate 2.

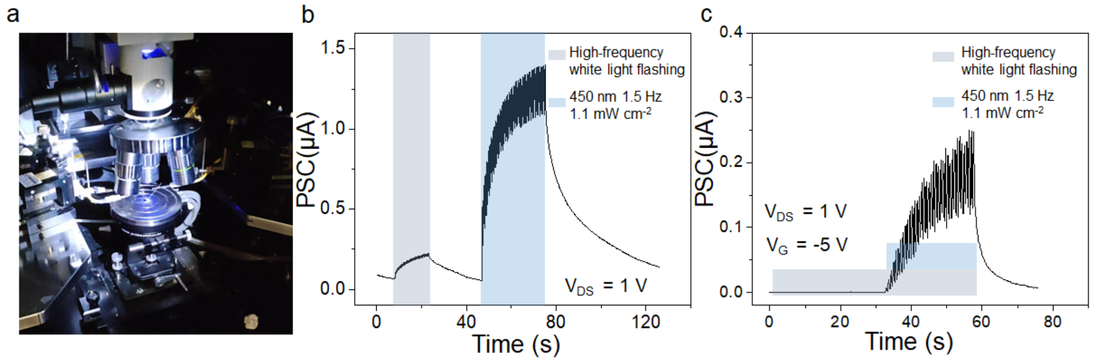

Supplementary Fig. 35| a, The photograph for noise filtering under mixed stimuli. **b**, Sequentially applied white light flashes and 450 nm laser irradiation. **c**, Apply a continuous white light flash at V_G = -5, followed by 450 nm laser illumination after a period of time. The device fabrication method employed here is consistent with that described in the main text, with a channel width of 5 μm.

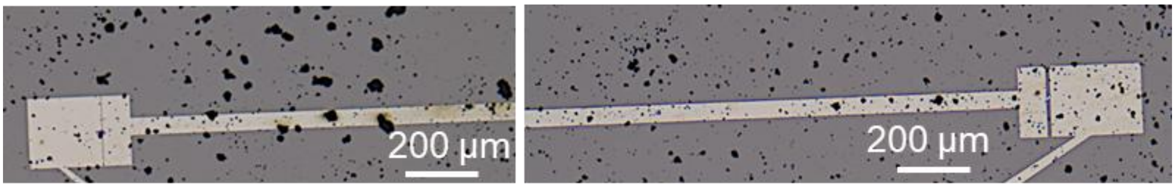

Supplementary Fig. 36| The optical images of two common-gate series devices with 1.5 μm and 10 μm channel lengths, respectively.

Supplementary references:

1 Juusola, M.; Hardie, R. C., Light adaptation in Drosophila photoreceptors: I. Response dynamics and signaling efficiency at 25 °C. *J. Gen. Physiol*. **2001**, 117 (1), 3-25.

2 de Ruyter van Steveninck, R. R.; Laughlin, S. B., The rate of information transfer at graded-potential synapses. *Nature* **1996**, 379 (6566), 642-645.

3 Li, D.; Meng, Y.; Zhang, Y.; Xie, P.; Zeng, Z.; Wang, W.; Lai, Z.; Wang, W.; Tsang, S.-W.; Wang, F.; Liu, C.; Lan, C.; Yip, S.; Ho, J. C., Selective Surface Engineering of Perovskite Microwire Arrays. *Adv. Funct. Mater*. **2023**, 33 (33), 2302866.
